# Circulating cardiac MicroRNAs safeguard against dilated cardiomyopathy

**DOI:** 10.1002/ctm2.1258

**Published:** 2023-05-03

**Authors:** Xiaolei Cheng, Dongdong Jian, Junyue Xing, Cihang Liu, Yong Liu, Cunying Cui, Zhen Li, Shixing Wang, Ran Li, Xiaohan Ma, Yingying Wang, Xiaoping Gu, Zhenwei Ge, Hao Tang, Lin Liu

**Affiliations:** ^1^ National Health Commission Key Laboratory of Cardiovascular Regenerative Medicine Heart Center of Henan Provincial People's Hospital Central China Fuwai Hospital of Zhengzhou University Fuwai Central China Cardiovascular Hospital and Central China Branch of National Center for Cardiovascular Diseases Zhengzhou China; ^2^ Department of Anesthesiology Affiliated Drum Tower Hospital of Medical School of Nanjing University Nanjing China; ^3^ Department of Biochemistry and Molecular Biology Beijing Key Laboratory of Protein Posttranslational Modifications and Cell Function School of Basic Medical Sciences Peking University Health Science Center Beijing China; ^4^ Henan Key Laboratory of Chronic Disease Management Department of Health Management Center Henan Provincial People's Hospital Department of Health Management Center of Central China Fuwai Hospital Central China Fuwai Hospital of Zhengzhou University Zhengzhou China; ^5^ Department of Physiology Shanxi Medical University Taiyuan China

**Keywords:** apoptosis, autophagy, circulating microRNA, dilated cardiomyopathy, FOXO3

## Abstract

**Background:**

Cardiac‐resident or ‐enriched microRNAs (miRNAs) could be released into the bloodstream becoming circulating cardiac miRNAs, which are increasingly recognized as non‐invasive and accessible biomarkers of multiple heart diseases. However, dilated cardiomyopathy (DCM)‐associated circulating miRNAs (DACMs) and their roles in DCM pathogenesis remain largely unexplored.

**Methods:**

Two human cohorts, consisting of healthy individuals and DCM patients, were enrolled for serum miRNA sequencing (10 vs. 10) and quantitative polymerase chain reaction validation (46 vs. 54), respectively. Rigorous screening strategy was enacted to define DACMs and their potentials for diagnosis. DCM mouse model, different sources of cardiomyocytes, adeno‐associated virus 9 (AAV9), gene knockout, RNAscope miRNA in situ hybridization, mRFP‐GFP‐LC3B reporter, echocardiography and transmission electron microscopy were adopted for mechanistic explorations.

**Results:**

Serum miRNA sequencing revealed a unique expression pattern for DCM circulating miRNAs. DACMs miR‐26a‐5p, miR‐30c‐5p, miR‐126‐5p and miR‐126‐3p were found to be depleted in DCM circulation as well as heart tissues. Their expressions in circulation and heart tissues were proven to be correlated significantly, and a combination of these miRNAs was suggested potential values for DCM diagnosis. FOXO3, a predicted common target, was experimentally demonstrated to be co‐repressed within cardiomyocytes by these DACMs except miR‐26a‐5p. Delivery of a combination of miR‐30c‐5p, miR‐126‐5p and miR‐126‐3p into the murine myocardium via AAV9 carrying an expression cassette driven by cTnT promoter, or cardiac‐specific knockout of FOXO3 (Myh6‐Cre^ERT2^, FOXO3 flox**^+/+^**) dramatically attenuated cardiac apoptosis and autophagy involved in DCM progression. Moreover, competitively disrupting the interplay between DACMs and FOXO3 mRNA by specifically introducing their interacting regions into murine myocardium crippled the cardioprotection of DACMs against DCM.

**Conclusions:**

Circulating cardiac miRNA‐FOXO3 axis plays a pivotal role in safeguarding against myocardial apoptosis and excessive autophagy in DCM development, which may provide serological cues for DCM non‐invasive diagnosis and shed light on DCM pathogenesis and therapeutic targets.

## 1 BACKGROUND

Dilated cardiomyopathy (DCM) is a non‐ischemic heart muscle disease with structural and functional myocardial abnormalities that could lead to substantial morbidity and mortality owing to complications such as heart failure and arrhythmia.^1^ DCM is clinically characterized by left or biventricular dilation and systolic dysfunction that are not explained by coronary artery disease, hypertension, valvular disease or congenital heart disease.^2^ Decades of research have revealed diverse etiologies for DCM, including genetic mutations, infections and inflammation, autoimmune deficiency, exposure to toxins and so on.^3^ The wide spectrum of DCM causes and its often‐slow progression to eventual signs and symptoms of heart failure hinder its early diagnosis and subsequent interventions to prevent irreversible remodeling and damage. Cellular and molecular changes are conceived to occur in the early stage of disease progression. Clearly, a dissection of the regulatory events shared by genetic and acquired DCM at the molecular level provides promising and valuable cues for DCM treatment. Hence, to date, several mechanistic insights have been reported to contribute to DCM pathologies, including alterations in force generation and transmission, disruption of energy production and consumption, altered metabolic profiles, aberrant autophagic processes and protein degradation, and abnormal calcium handling.^4^, ^5^, ^6^, ^7^, ^8^ These diverse mechanisms in DCM are suggested to lead to a final common pathway, with the outcome of cardiac cell death, leading to left ventricular systolic dysfunction and heart failure.^9^

MicroRNAs (miRNAs) constitute a class of ∼22 nt regulatory non‐coding RNAs that post‐transcriptionally regulate gene expression, leading to mRNA degradation or translational inhibition of targeted transcripts.^10^ Numerous studies have suggested that miRNAs play important roles in cardiovascular development and diseases.^11^, ^12^ Altered miRNA expression and reactivation of fetal miRNA program have been previously suggested to affect the human DCM heart.^13^, ^14^ Moreover, global loss of miRNA‐mediated regulation in the murine heart via deletion of dicer or dgcr8, both of which are required for miRNA processing and biogenesis, has been reported to lead to DCM and heart failure.^15^, ^16^ Despite these findings, detailed mechanisms of miRNA actions in regulating DCM progression remain largely unclear. MiRNAs are usually released into the bloodstream where they are present in concentration levels that differ between healthy subjects and diseased patients, and in turn act in a systemic mode beyond a single tissue.^11^ Accumulating evidence indicates that circulating miRNAs exhibit great potential as non‐invasive and readily accessible biomarkers for risk stratification, diagnosis and prognosis of multiple forms of cardiovascular diseases.^17^, ^18^, ^19^ In this regard, screening DCM‐associated circulating microRNAs (DACMs), particularly those residing or enriched within the myocardium, and elaborating on their actions appear to be feasible avenues to provide mechanistic understanding of the role of miRNAs in DCM development and urgently needed for the development of miRNA‐based clinical applications and therapeutics for DCM.

In this study, we profiled circulating miRNAs in DCM patients by serum miRNA sequencing and established a set of circulating miRNAs (miR‐30c‐5p, miR‐126‐5p and miR‐126‐3p) intimately correlated with DCM pathogenesis. Our results suggest that cardiac replenishment of these miRNAs significantly reduced myocardial apoptosis and excessive autophagy, and in turn ameliorated DCM progression. Mechanistically, miR‐30c‐5p, miR‐126‐5p and miR‐126‐3p co‐suppress cardiac FOXO3 expression, and loss of them abrogates their repression on FOXO3 thus aggravating cardiac apoptosis and autophagy in DCM development. Taken together, these findings may provide serological clues for microRNA‐based non‐invasive diagnosis of DCM in the future and shed new light on DCM pathogenesis and associated therapeutic targets.

## 2 MATERIALS AND METHODS

Detailed materials and methods are available in the Supplemental Information that can be found with this article online.

### 2.1 Statistical analysis

Statistical analysis was performed using commercial software (SPSS 24, Chicago, IL, USA). For clinical data, parametric (data followed normal distribution, independent two‐sided student's *t*‐test) and non‐parametric tests (data didn't follow normal distribution, independent samples Kruskal‐Wallis test) were used as appropriate. For experimental data, normality was assessed using Shapiro‐Wilk test. One‐way analysis of variance was used to evaluate the differences among groups followed by a post Tukey test, or two‐sided student's *t*‐test was employed for the comparison between the two groups. For ROC (receiver operating characteristic) analysis, the AUC (area under curve, 95% CI) was calculated based on ROC curves. All quantitative data were presented as mean ± standard deviation. The significance threshold was set at *p* < 0.05.

## 3 RESULTS

### 3.1 Profiling of circulating MicroRNAs closely associated with DCM

**FIGURE 1:**
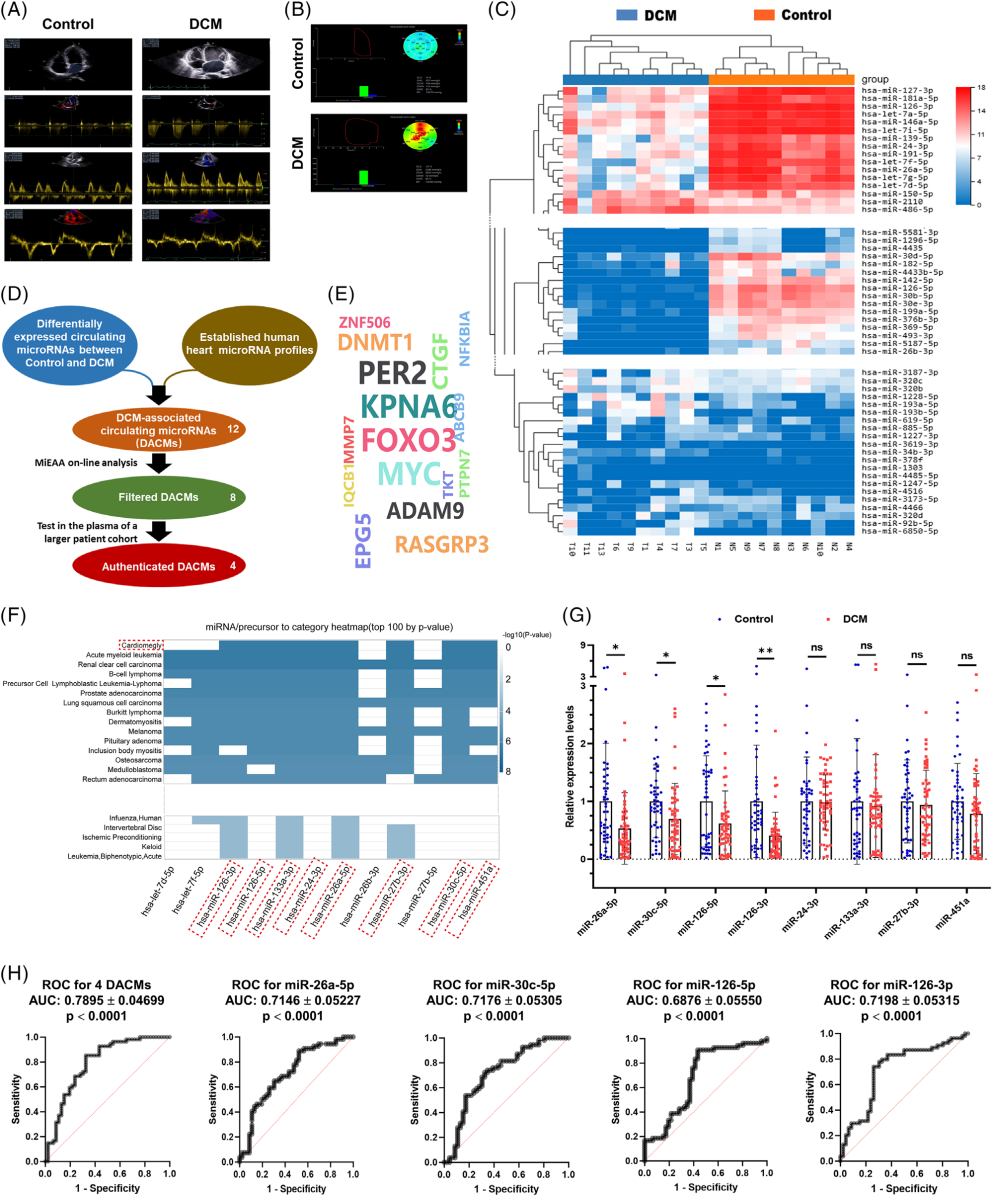
**Profiling of circulating microRNAs (miRNAs) closely associated with dilated cardiomyopathy (DCM). (A and B)** Echocardiographic evaluation of DCM patients (DCM) and healthy control persons (Control). (**C)** Heatmap of circulating miRNA expressions in DCM patients (DCM) and healthy control persons (Control). (**D)** Schematic description of the strategy utilized for the screening of DCM‐associated circulating miRNAs (DACMs). (**E)** Target prediction of DACMs by online analysis tool MiEAA (https://ccb‐compute2.cs.uni‐saarland.de/mieaa2/). (**F)** Enrichment analysis of DACMs by MiEAA. (**G)** Quantitative polymerase chain reaction (qPCR) validation of the eight candidate DACMs closely associated with cardiomegaly in the serum of a larger human cohort (Control vs. DCM, 46 vs. 54). (**H)** ROC (receiver operating characteristic) curve analysis was performed by SPSS software to assess the diagnostic performance of miR‐26a‐5p, miR‐30c‐5p, miR‐126‐5p and miR‐126‐3p. The AUC (95% CI) was calculated based on ROC curves. *p < 0.05; **p < 0.01; ns, no significance.

To profile DCM‐associated circulating microRNAs, human serum procured from 10 DCM patients (DCM group), who had been accurately diagnosed by echocardiography (Figure 1A,B, Figure S1A–C), and 10 healthy control ones (Control group) with comparable parameters (Table 1) was prepared for miRNA sequencing. Clustering (Figure 1C) and correlation (Figure S1D) analysis showed that circulating miRNAs were differentially expressed between control and DCM groups, hinting at a potential for use in DCM diagnosis. Next, we stepwise refined the repertoire of DACMs as illustrated in Figure 1D. Twelve typical miRNAs (Table S1) were obtained after cross‐referencing the differentially expressed circulating miRNAs with cardiac miRNAs identified to date.^12^, ^13^, ^14^, ^16^ MiEAA (https://ccb‐compute2.cs.uni‐saarland.de/mieaa2/), an online analysis tool,^20^ predicted a plethora of mRNA targets of these miRNAs (Figure 1E and Table S2), among which eight were highlighted to be associated with cardiomegaly (Figure 1F). To authenticate the expression changes of the eight miRNAs in the circulation, quantitative polymerase chain reaction (qPCR) validation was performed in quick succession on a larger cohort (control vs. DCM, 46 vs. 54, Table 2). As shown in Figure 1G, a significant reduction in serum levels was observed only for miR‐26a‐5p, miR‐30c‐5p, miR‐126‐5p and miR‐126‐3p, distinct from the miRNA sequencing data, and this could be attributed to individual discrepancies especially when the sample volume here used for miRNA sequencing was limited. Furthermore, we performed ROC curve analysis to determine their diagnostic potentials for DCM. Our data suggested that a combination of these four DACMs possessed an obvious superiority in DCM diagnosis to each one alone (Figure 1H) or any other two or three combinations (data unpublished). Altogether, DCM is able to be well‐characterized by the expression pattern of circulating microRNAs, some of which owning a great abundance in the heart may be advantageous candidates for DCM non‐invasive diagnostic biomarkers.

**TABLE 1:** Baseline characteristics of the cohort used for microRNA sequencing.

Parameter	Control (*n* = 10)	DCM patients (*n* = 10)	*p*‐Value
Anthropometrics data			
Age (years)	43.90 ± 9.67	39.00 ± 8.42	0.216
Male sex, *n*	4	5	0.653
BMI (Kg/m^2^)	23.05 ± 2.02	23.76 ± 1.88	0.424
Tobacco use, *n*	1	1	1.000
Resting heart rate (b.p.m.)	68.10 ± 7.13	76.50 ± 8.82	0.031
Systolic blood pressure (mmHg)	117.90 ± 10.45	103.70 ± 8.31	0.003
Diastolic blood pressure (mmHg)	71.30 ± 7.65	58.20 ± 16.71	0.026
Metabolic status			
Total cholesterol (mmol/L)	4.42 ± 0.70	4.59 ± 0.87	0.628
Triglycerides (mmol/L)	1.55 ± 0.37	2.45 ± 1.63	0.104
Creatinine (mg/dL)	60.00 ± 14.59	94.10 ± 53.49	0.068
Haemoglobin (g/L)	128.30 ± 10.88	138.30 ± 25.81	0.274
Glucose (mmol/L)	4.84 ± 0.54	5.07 ± 0.61	0.380
NT‐proBNP	44.60 ± 30.69	2851.30 ± 1532.12	<0.001
Drug therapy			
ACEI/ARB, *n*	1	9	<0.001
Beta‐blocker, *n*	0	10	<0.001
Aldosterone antagonist, *n*	0	10	<0.001
Loop diuretic, *n*	0	10	<0.001
SGLT2i, *n*	1	5	0.051
Left ventricle			
End‐diastolic volume (mL)	108.00 ± 10.88	221.50 ± 46.60	<0.001
End‐systolic volume (mL)	39.70 ± 4.69	150.30 ± 42.89	<0.001
Stroke volume (mL)	71.50 ± 6.98	59.80 ± 4.69	<0.001
Ejection fraction (%)	65.60 ± 1.27	39.30 ± 10.70	<0.001
Mass (g)	158.42 ± 36.31	197.97 ± 29.91	0.016
NYHA functional class			
Class I or II, *n*	–	2	–
Class III or IV, *n*	–	8	–
Concurrent diseases			
Hypertension	0	0	–
Coronary heart disease	0	0	–
Atrial fibrillation	0	1	0.305
COPD	0	0	–
Diabetes	0	0	–
Stroke or TIA	0	0	–
Cancers	0	0	–
DCM duration (years)	–	1.67 ± 2.03	–

Abbreviations: ACEI/ARB, angiotensin converting enzyme inhibitor (ACEI) and angiotensin receptor antagonist (ARB); BMI, body mass index; DCM, dilated cardiomyopathy; SGLT2i, sodium‐dependent glucose transporters 2 inhibitor; TIA, transient ischemic attacks.

**TABLE 2:** Baseline characteristics of the larger cohort for testing.

Parameter	Control (*n* = 46)	DCM patients (*n* = 54)	*p*‐Value
Anthropometrics data			
Age (years)	42.72 ± 9.02	40.44 ± 10.33	0.248
Male sex, *n* (%)	31 (67.39%)	43 (79.63%)	0.164
BMI (Kg/m^2^)	22.93 ± 1.78	23.57 ± 1.67	0.066
Tobacco use, *n* (%)	12 (26.09%)	10 (18.52%)	0.363
Resting heart rate (b.p.m.)	73.83 ± 6.06	80.61 ± 8.99	<0.001
Systolic blood pressure (mmHg)	118.30 ± 8.91	111.48 ± 12.00	0.002
Diastolic blood pressure (mmHg)	73.70 ± 4.12	70.37 ± 12.05	0.069
Metabolic status			
Total cholesterol (mmol/L)	4.26 ± 0.57	4.06 ± 0.80	0.149
Triglycerides (mmol/L)	1.93 ± 0.76	1.63 ± 0.93	0.084
Creatinine (mg/dL)	66.63 ± 14.70	79.20 ± 39.21	0.017
Haemoglobin (g/L)	137.28 ± 15.86	136.56 ± 21.00	0.848
Glucose (mmol/L)	4.94 ± 0.71	5.24 ± 1.07	0.114
NT‐proBNP (pg/ml)	36.96 ± 20.48	2067.76 ± 1797.62	<0.001
Drug therapy			
ACEI/ARB, *n* (%)	3 (6.52%)	49 (90.74%)	<0.001
Beta‐blocker, *n* (%)	3 (6.52%)	51 (94.44%)	<0.001
Aldosterone antagonist, *n* (%)	0	52 (96.30%)	<0.001
Loop diuretic, *n* (%)	2 (4.35%)	54 (100%)	<0.001
SGLT2i, *n* (%)	2 (4.35%)	42 (77.78%)	<0.001
Left ventricle			
End‐diastolic volume (mL)	114.52 ± 17.25	240.96 ± 50.83	<0.001
End‐systolic volume (mL)	39.89 ± 8.50	170.40 ± 47.09	<0.001
Stroke volume (mL)	71.67 ± 9.61	62.44 ± 10.79	<0.001
Ejection fraction (%)	64.98 ± 3.17	31.46 ± 11.58	<0.001
Mass (g)	156.25 ± 31.16	192.13 ± 46.15	<0.001
NYHA functional class			
Class I or II, *n* (%)	–	12 (22.22%)	–
Class III or IV, *n* (%)	–	42 (77.78%)	–
Concurrent diseases			
Hypertension	3 (6.52%)	0	0.057
Coronary heart disease	2 (4.35%)	1 (1.85%)	0.655
Atrial fibrillation	0	4 (7.41%)	0.060
COPD	1 (2.17%)	0	0.276
Diabetes	2 (4.35%)	2 (3.70%)	0.870
Stroke or TIA	0	1 (1.85%)	0.354
Cancers	1 (2.17%)	0	0.276
DCM duration (years)	–	1.34 ± 2.20	–

Abbreviations: ACEI/ARB, angiotensin converting enzyme inhibitor (ACEI) and angiotensin receptor antagonist (ARB); BMI, body mass index; DCM, dilated cardiomyopathy; SGLT2i, sodium‐dependent glucose transporters 2 inhibitor; TIA, transient ischemic attacks.

### 3.2 DACM expression in circulation positively correlates with that in the heart

**FIGURE 2:**
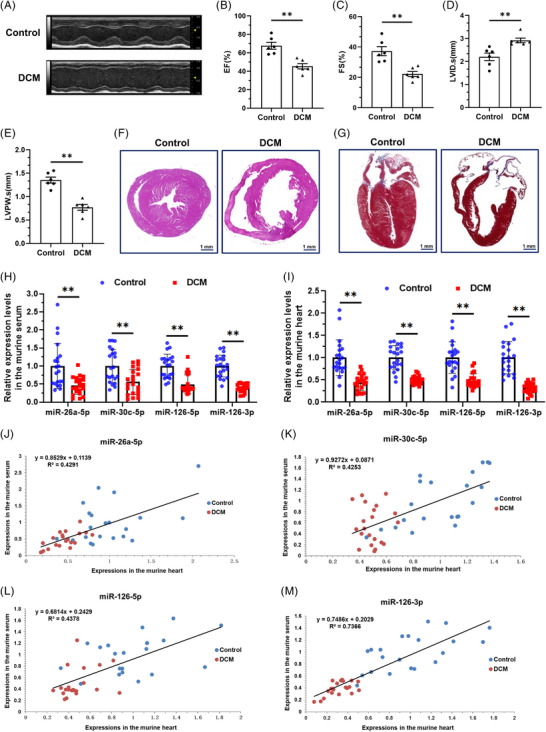
**Dilated cardiomyopathy (DCM)‐associated circulating miRNAs (DACMs) expression in circulation positively correlates with that in the heart. (A–E)** Characterization of DCM manifestations by echocardiography in the established murine model. EF, left ventricular ejection fraction; FS, left ventricular fraction shortening; LVID, left ventricular diameter at end‐systole (s); LVPW, left ventricular posterior wall thickness at end‐systole (s). (**F and G)** Representative pictures of the histological staining of murine hearts. F, hematoxylin‐eosin (H&E) staining; G, Masson staining. Scale bar, 1 mm. (**H** and **I)** Quantitative PCR analysis of the expression levels of the four established DACMs in the murine serum (H) and heart tissue (I) (Control vs. DCM, 21 vs. 21). (**J–M)** Correlation analysis of DACM expressions in the murine serum and heart tissue. J, miR‐26a‐5p, r (pearson correlation coefficient) = 0.655, *p* < 0.01; K, miR‐30c‐5p, r = 0.652, *p* < 0.01; L, miR‐126‐5p, r = 0.662, *p* < 0.01; M, miR‐126‐3p, r = 0.858, *p* < 0.01; **p < 0.01.

To uncover the interrelationship of the four DACMs with DCM, we firstly probed into their expressive correlation between the circulation and the heart. MicroRNAs are thought to be highly conserved in sequence and function among species.^21^ Indeed, in humans, rats, and mice, the four DACMs harbor identical sequences (Figure S2A). Considering this together with the unavailability of human heart tissues, a doxorubicin‐induced DCM mouse model (Supplementary Figure 2B)^22^ was employed to investigate the association of DACMs with DCM pathogenesis. These modeling mice were well‐characterized with DCM manifestations (Figure 2A–G), followed by RNA isolation of the serum and heart tissues to evaluate DACM expression. Consistent with the changes in human serum level, an expressive defect of four DACMs was significantly noted in DCM murine serum (Figure 2H) as well as heart tissues (Figure 2I). To attempt to extend this finding to human hearts, publicly available miRNA data from human failing and healthy control hearts (documented in a previous study^14^) were reanalyzed. In sharp contrast with its decrease in the circulation of DCM patients, miR‐26a tended to be up‐regulated in the failing human hearts, whereas both levels of miR‐30c and miR‐126 (miR‐126‐5p and miR‐126‐3p) demonstrated a tendency to decrease (Figure S2C), similar to their serological changes on DCM patients. This discrepancy could be explained by the diversity of etiologies leading to the failing heart that is not confined to DCM. Subsequently, correlation analysis pointed out that the expressions of four DACMs in DCM murine blood were all significantly correlated with those in the heart tissues in a positive way (Figure 2J–M), strongly indicative of an interconnection between serological and cardiac miRNAs. Jointly, these data suggest that miR‐26a‐5p, miR‐30c‐5p, miR‐126‐5p and miR‐126‐3p are four conserved circulating miRNAs closely associated with DCM, and their altered expressions may indicate and contribute to DCM progression.

### 3.3 FOXO3 – A common target of DCM‐associated circulating MicroRNAs

**FIGURE 3:**
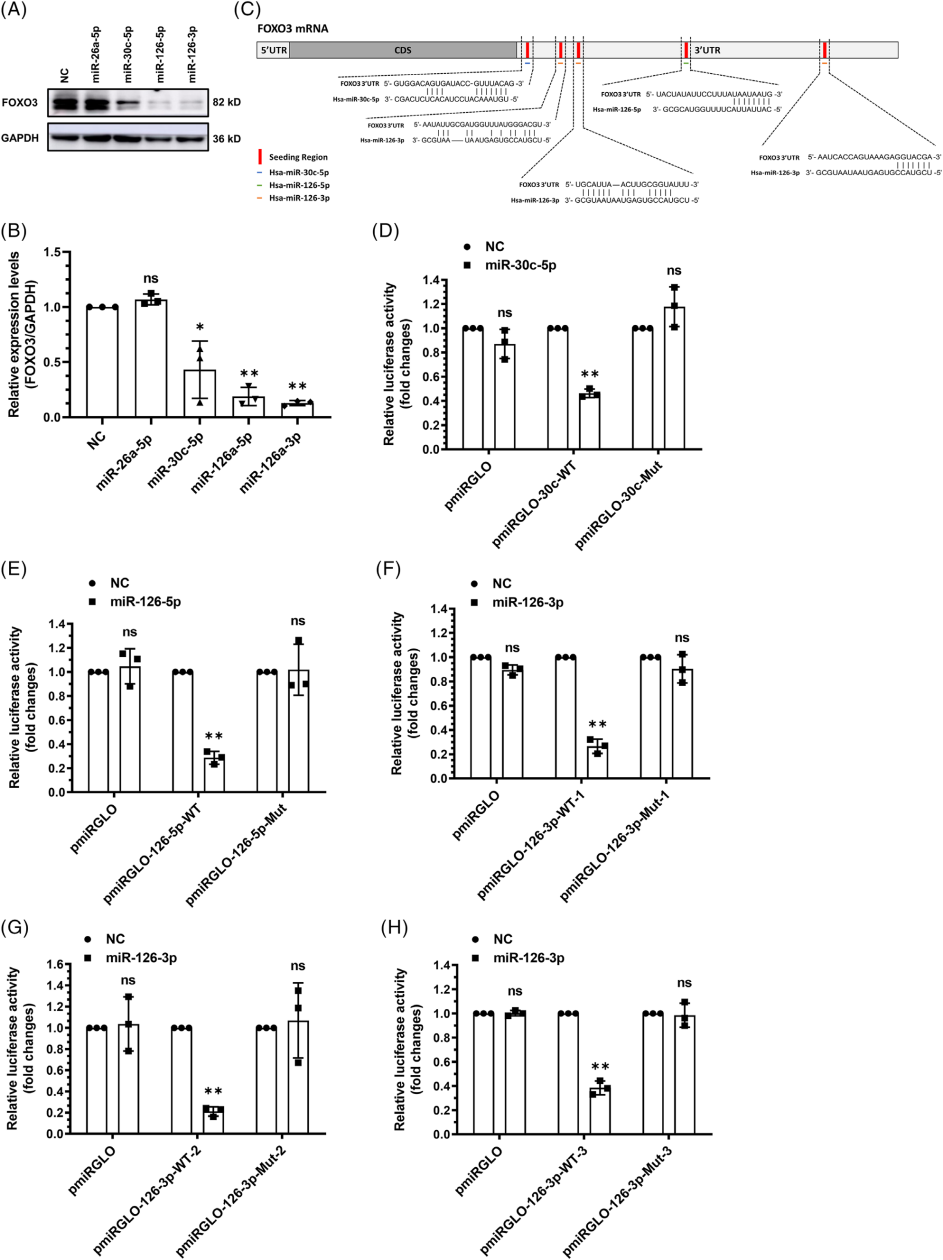
**FOXO3 ‐ an important shared target of** **dilated cardiomyopathy (DCM)** **‐associated circulating microRNAs (DACMs). (A)** Immunoblotting analysis of FOXO3 expression in AC16 cells transfected with negative control (NC) or DACM mimics. GAPDH served as a loading control. (**B)** The density of the signals in A was scanned and plotted as the means ± SD from three independent experiments. (**C)** Schematic description of the predicted binding sites of DACMs. (**D–H)** Dual luciferase activity was determined in AC16 cells transfected with different reporter constructs in the presence of DACM or not. pmiRGLO, control reporter construct; pmiRGLO‐miR‐WT, reporter construct bearing the acting regions of involved microRNA; pmiRGLO‐miR‐Mut, reporter construct bearing mutations in the acting regions of involved microRNA; Data represent as mean ± SD from three independent experiments. *p < 0.05; **p < 0.01; ns, no significance.

To dissect the regulatory role of identified four DACMs in DCM development, we focused on their mRNA targets. According to the prediction analysis (Table S2), FOXO3, well‐documented in governing cellular apoptosis and autophagy,^23^, ^24^, ^25^ was conspicuously proposed as a latent common target gene. To confirm this, miR‐26a‐5p, miR‐30c‐5p, miR‐126‐5p and miR‐126‐3p mimics were separately transfected into AC16 (immortalized human ventricular myocytes) cells (Figure S3A). Intriguingly, immunoblot results showed that these miRNAs effectively repressed FOXO3 expression except miR‐26a‐5p (Figure 3A and 3B), implying that miR‐26a‐5p cannot truly act on FOXO3 mRNA. Given this, we next mainly focused on the other three DACMs (miR‐30c‐5p, miR‐126‐5p and miR‐126‐3p) and then mapped their potential action sites on FOXO3 mRNA via in silico analysis as illustrated in Figure 3C. Of note, miR‐126‐3p was predicted to act on three dispersed regions within FOXO3 mRNA. To verify that these regions were key to the repressive action of DACMs on FOXO3 expression, dual luciferase reporter constructs bearing wild‐type or mutant seed regions for DACM action were generated, and then co‐transfected with DACM mimics into AC16 cells, followed by the determination of luciferase activity. Upon overexpression of these miRNAs (Figure S3B–D), a marked decrease in luciferase activity was observed merely in the cells transfected with wild‐type reporter constructs, but not in those transfected with control or mutant reporter constructs (Figure 3D–H), corroborating the status of these sites as miRNA acting interface. Altogether, these observations suggest that miR‐30c‐5p, miR‐126‐5p and miR‐126‐3p are all able to act on FOXO3 mRNA thereby simultaneously restraining its expression.

### 3.4 Co‐suppression of FOXO3 expression by DACMs occurs within the cardiomyocyte

**FIGURE 4:**
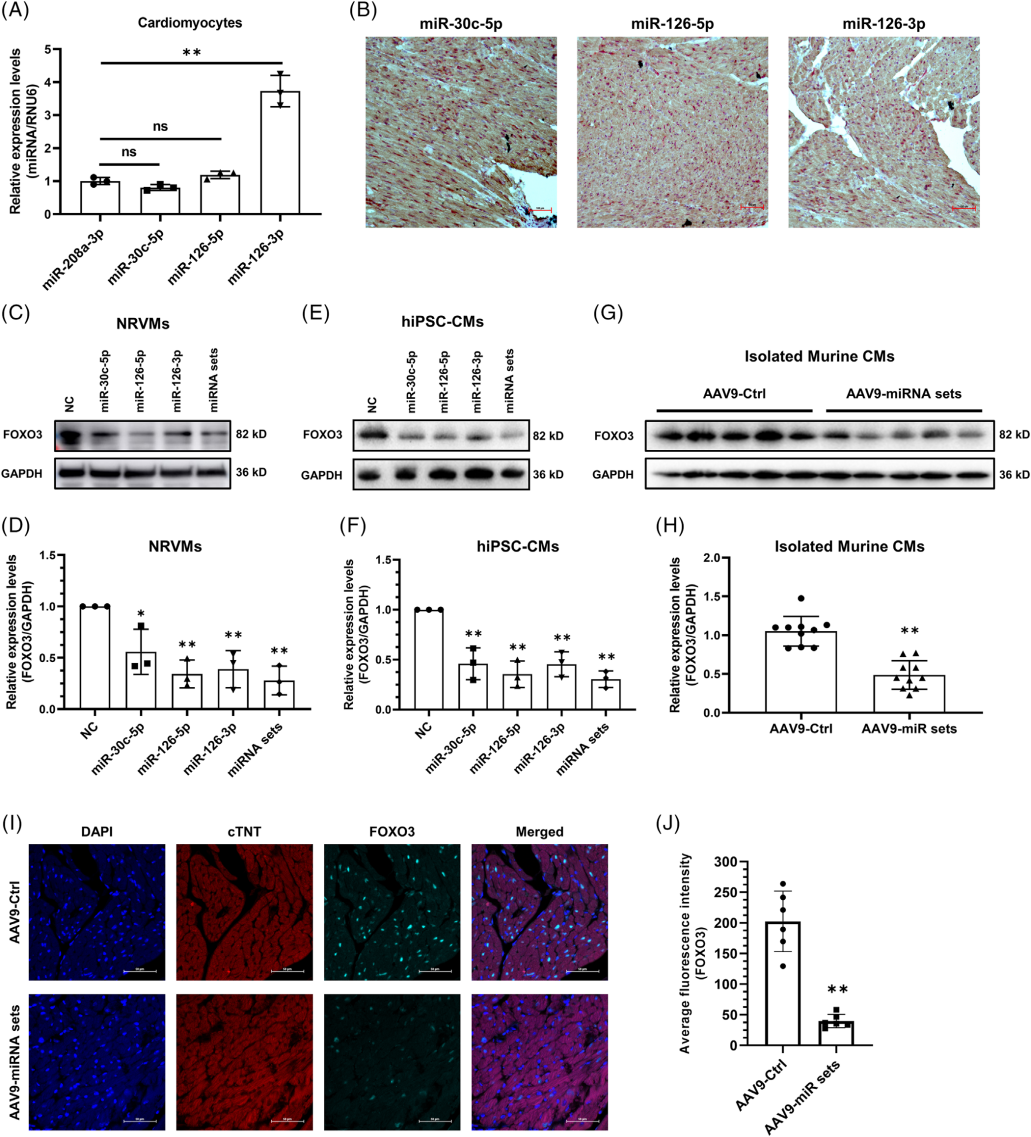
**Co‐suppression of FOXO3 by** **dilated cardiomyopathy (DCM)‐associated circulating miRNAs (DACMs)** **occurs within the cardiomyocyte. (A)** Relative expression levels of microRNA (miRNA) assessed by quantitative polymerase chain reaction (qPCR) in isolated murine ventricular myocytes.^31^ (**B)** In situ RNA hybridization with RNAscope probes to analyze the expression of DACMs in human ventricular tissues. Nuclei, hematoxylin (blue); cardiomyocytes, cardiac troponin T (cTnT, brown); DACMs (red dots). Scale bar, 100 μm. (**C)** Immunoblotting analysis of FOXO3 expression in neonatal rat ventricular myocytes (NRVMs) infected with adenoviruses expressing negative control (NC) or DACM mimics. miRNA sets, a combination of DACMs. GAPDH served as a loading control. (**D)** The density of the signals in C was scanned and plotted as the means ± SD from three independent experiments. (**E)** Immunoblotting analysis of FOXO3 expression in human induced pluripotent stem cell‐derived cardiomyocytes (hiPSC‐CMs) infected with adenoviruses expressing negative control (NC) or DACM mimics. miRNA sets, a combination of DACMs. GAPDH served as a loading control. (**F)** The density of the signals in E was scanned and plotted as the means ± SD from three independent experiments. (**G)** Immunoblotting analysis of FOXO3 expression in cardiac myocytes isolated by Langendorff from mice infected with adeno‐associated virus serotype 9 (AAV9). AAV9‐Ctrl, control viruses; AAV9‐miRNA sets, DACM‐expression viruses. GAPDH served as a loading control. (**H)** The density of the signals in G was scanned and plotted as the means ± SD, *n* = 10 for each group. (**I)** Representative immunofluorescence images of heart tissue sections from mice described in G. Nuclei, DAPI (blue); cardiomyocytes, cardiac troponin T (cTnT, red); CIRBP (cyan). Scale bar, 50 μm. (**J)** Quantification of the relative FOXO3 fluorescence intensity in six microscopic fields from three hearts per group in I. *p < 0.05; **p < 0.01.

Increasing evidence indicates that miRNAs could act in a systemic mode, not confined to a specific cellular compartment, to regulate gene expressions, especially in tissues and organs consisting of miscellaneous types of cells.^11^ For instance, miR‐126, a well‐characterized pro‐angiogenic miRNA encoded by an intron of endothelial‐specific gene Egfl7,^26^, ^27^, ^28^ has been reported to modulate platelet function and to promote leukemogenesis.^29^, ^30^ To test if the co‐suppression of FOXO3 expression by DACMs does occur within the cardiomyocyte, we firstly scrutinized their existence in it. Physical isolation of purified adult murine cardiac myocytes was performed according to the method described in a previous work.^31^ Cardiomyocyte purity was verified by the expression of the marker genes cTnT (cardiomyocytes) and COL1A1 (non‐cardiomyocytes, the remanent cell mixtures after cardiomyocyte isolation) (Figure S4A). Quantitative PCR results showed that DACMs were expressed at a level comparable (miR‐30c‐5p and miR‐126‐5p) to or even higher (miR‐126‐3p, ∼3.73 folds) than cardiac‐enriched myomiR miR‐208a^32^ in isolated cardiomyocytes (Figure 4A), indicative of a relatively high abundance of DACMs in the myocardium. Furthermore, an in situ RNAscope‐based hybridization analysis of DACMs was performed in the human ventricular tissues. As displayed in Figure 4B, three DACMs (red dots) clearly localized within the stained myocytes (cTnT, brown), demonstrating the presence of DACMs in the cardiomyocytes. Subsequently, we investigated the repressive action of DACMs on FOXO3 expression in myocardial cells among different species, including humans, rats and mice. Neonatal rat ventricular myocytes (NRVMs) and human induced pluripotent stem cell‐derived cardiomyocytes (hiPSC‐CMs) were infected with adenoviruses carrying different DACM expression cassettes. Consistent with the results observed within AC16 cells, DACM overexpression, either alone or in combination, successfully reduced FOXO3 expression in both NRVMs (Figure 4C and 4D) and hiPSC‐CMs (Figure 4E and 4F). In addition, adeno‐associated virus serotype 9 (AAV9) bearing DACM‐expressing cassettes driven by the cTnT promoter (pre‐miR‐30c and pre‐miR‐126 expression cassettes are under control of two separate cTnT promoters but are present in one virus packaging construct) was leveraged to specifically overexpress DACMs in the murine myocardium (Figure S4B). The three DACMs were proven to be markedly overexpressed in Langendorff isolated cardiomyocytes (Figure S4C). As well, DACMs suppressed mouse myocardial expression of FOXO3, which could be evidenced by the marked decrease in FOXO3 protein levels in the cardiac myocytes (Figure 4G,H) and the attenuation of FOXO3 fluorescence intensity within the myocardium (Figure 4I,J). Based on these data, we concluded that DACM‐mediated co‐suppression of FOXO3 expression was evident within cardiomyocytes and appeared to be relatively conserved among species.

### 3.5 DACMs attenuate myocardial apoptosis and autophagy in DCM progression

**FIGURE 5:**
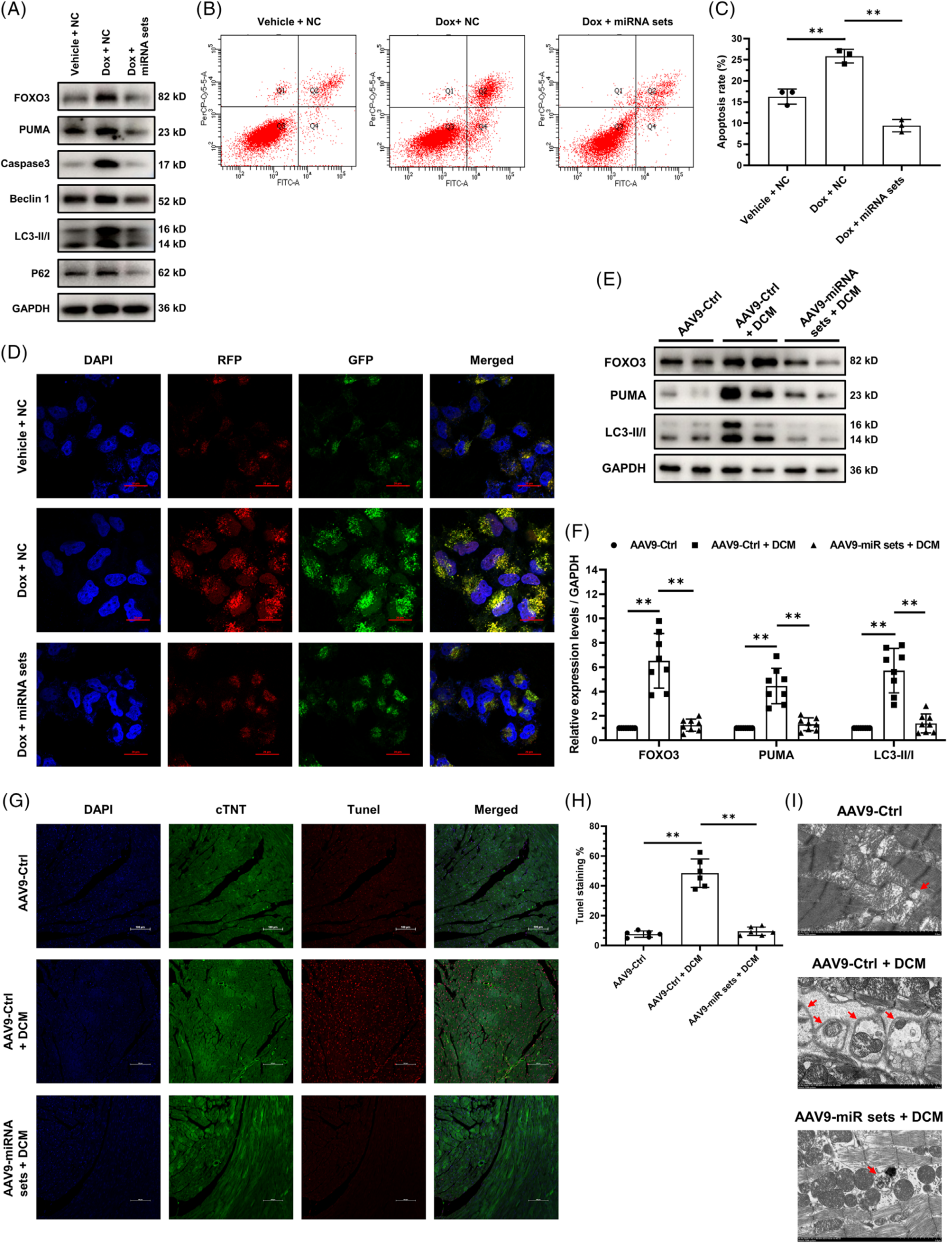
**Dilated cardiomyopathy (DCM)‐associated circulating miRNAs (DACMs)** **attenuate myocardial apoptosis and autophagy in the development of DCM. (A)** Immunoblotting analysis of the Expression of FOXO3, PUMA, Caspase3 (cleaved), Beclin1, LC3‐II/I and P62 in AC16 cells transfected with negative control (NC) or DACM mimics in the presence of doxorubicin (Dox) treatment (300 nM for 24 h) or not. GAPDH served as a loading control. (**B and C)** Representative images of flow cytometry (B) and statistical analysis of apoptosis (C) of AC16 cells described in A. (**D)** Representative fluorescence images of AC16 reporter cells (stably expressing mRFP‐GFP‐LC3B reporter protein to assess autophagy process) subjected to identical manipulations as described in A. Nuclei, DAPI (blue); mRFP‐LC3B (indicating total LC3B, red); GFP‐LC3B (indicating unacidified LC3B, green); mRFP‐GFP‐LC3B (indicating unacidified LC3B, yellow). Scale bar, 20 μm. (**E)** Immunoblotting analysis of the expression of FOXO3, PUMA and LC3‐II/I in the heart tissues from mice receiving adeno‐associated virus serotype 9 (AAV9) infection and (or) DCM induction. AAV9‐Ctrl, control viruses; AAV9‐miRNA sets, DACM‐expression viruses. GAPDH served as a loading control. (**F)** The density of the signals in E was scanned and plotted as the means ± SD, *n* = 10 for each group. (**G)** TUNEL staining was used to assess murine cardiac apoptosis. Nuclei, DAPI (blue); cardiomyocytes, cardiac troponin T (cTnT) (green); TUNEL (red). Scale bar, 100 μm. (**H)** Quantification of the TUNEL staining in six microscopic fields from three hearts per group in G. (**I)** Representative transmission electron microscopic images of the murine heart tissue sections. Red arrow indicated the autophagosome. Scale bar, 2 μm. **p < 0.01.

FOXO3 has been documented to activate the transcription of genes encoding BNIP3, PUMA, Beclin1 and LC3B, thereby contributing to cellular apoptosis and autophagy.^25^, ^33^, ^34^, ^35^, ^36^ Both the biological processes have been reported to be involved in DCM pathogenesis.^3^ Therefore, we investigated the influence of DACMs on myocardial apoptosis and autophagy in DCM development. At the cellular level, DACM (miRNA sets) administration significantly repressed Dox‐induced increases in FOXO3, PUMA, Caspase3 (cleaved), Beclin1, LC3‐II/I (LC3B) and P62 levels in AC16 cells (Figure 5A). Meanwhile, we evaluated cellular apoptosis by flow cytometry. As shown in Figure 5B and 5C, transfection of DACMs remarkably dampened Dox‐induced apoptosis of AC16 cells. In regards to autophagy, AC16 reporter cells stably expressing mRFP‐GFP‐LC3B were established. Compared to control cells (Vehicle + NC), autophagy initiation was identified to be enhanced overtly in the reporter cells subjected to Dox treatment (Dox + NC), but the autophagic flux appeared none accelerated because of the complete overlay of RFP and GFP signals, which could likely be attributed to lysosome acidification impairment led by Dox.^37^ However, DACM overexpression (Dox + miRNA sets) significantly mitigated the autophagic process in reporter cells (Figure 5D). Moreover, we examined the effect of DACMs against DCM‐related apoptosis and autophagy in the murine heart. Mice were infected with the above‐mentioned AAV9 viruses expressing DACMs 30 days before Dox administration (Supplementary Figure 5A). Cardiac‐specific overexpression of DACMs did not affect the cardiac function at baseline (Figure S5B–D). The protein expression levels of FOXO3, PUMA, and LC3‐II/I were markedly elevated in DCM murine hearts, suggesting the activation of both apoptosis and autophagy (Figure 5E,F). Replenishment of the three DACMs in the murine myocardium (Figure S5E) almost completely abrogated the expressive elicitation of FOXO3, PUMA and LC3‐II/I (Figure 5E,F). In addition, TUNEL (terminal‐deoxynucleoitidyl transferase mediated nick end labeling) staining (Figure 5G,H) and transmission electron microscopic imaging (Figure 5I) of murine heart tissues reiterated the potency of DACMs in reducing cardiomyocyte apoptosis and autophagy involved in DCM progression, respectively. In sum, these findings demonstrate that DACMs safeguard myocardium against DCM‐associated apoptosis and excessive autophagy.

### 3.6 DACMs ameliorate the development of DCM

**FIGURE 6:**
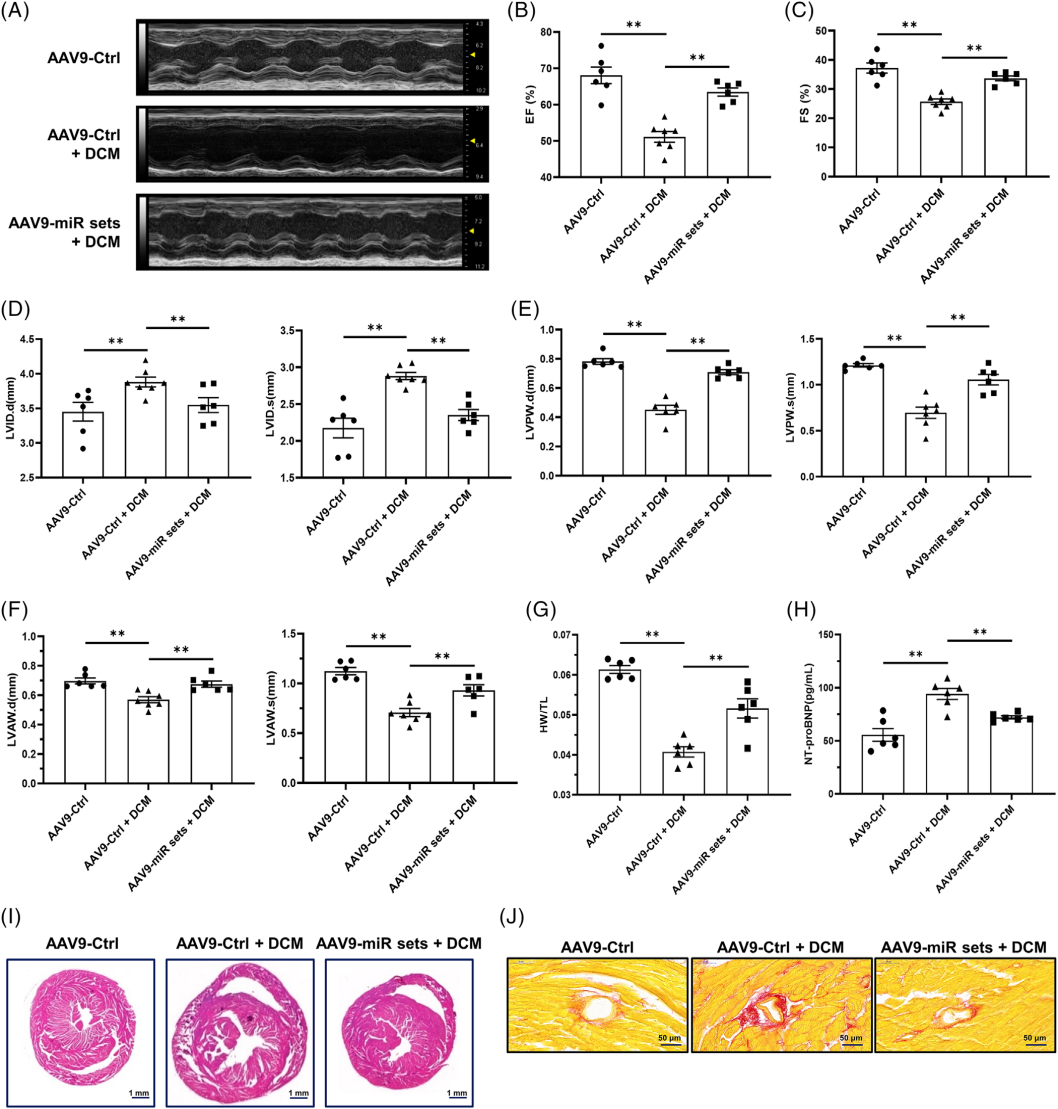
**Dilated cardiomyopathy (DCM)‐associated circulating miRNAs (DACMs) ameliorate DCM pathological manifestations. (A)** Representative echocardiographic images of mice described in Figure 4E. (**B–F)** Echocardiography evaluation for murine left ventricular ejection fraction (B, EF), left ventricular fractional shortening (C, FS), left ventricular internal diameter at end‐diastole and end‐systole (D, left, LVID.d; right, LVID.s), left ventricular posterior wall thickness at end‐diastole and end‐systole (E, left, LVPW.d; right, LVPW.s), left ventricular anterior wall thickness at end‐diastole and end‐systole (F, left, LVAW.d; right, LVAW.s), *n* = 6–7. (**G)** Statistical analysis of murine heart weight/tibia length (HW/TL) ratio, *n* = 6–7. (**H)** Determination of murine serum NT‐proBNP levels by ELISA, *n* = 6–7. (**I and J)** Representative histological staining images of heart tissue sections from mice described in A. I, hematoxylin‐eosin (H&E) staining, scale bar, 1 mm. J, Picrosirius red staining, scale bar, 50 μm. **p < 0.01.

In the meantime, an overall determination of DCM development in the model mice was performed. Notably, the impaired cardiac function identified in DCM mice was largely ameliorated after the administration of DACMs, as manifested by the improved motion of the left ventricular wall (Figure 6A) and maintained EF and FS values (AAV9‐Ctrl: EF, 68.07 ± 5.62%; FS, 37.22 ± 4.23%; AAV9‐Ctrl + DCM: EF, 51.14 ± 3.90%; FS, 25.68 ± 2.44%; AAV9‐miR sets + DCM: EF, 63.47 ± 2.83%; FS, 33.70 ± 2.06%; Figure 6B,C). DCM progression is marked by obvious morphological changes of the ventricle, including enlargement of the chamber as well as thinning of the ventricular wall. AAV9‐miR set injection successfully reversed these adverse structural alterations in murine hearts (Figure 6D–F), underscoring the substantial role DACMs played in safeguarding against DCM‐associated cardiac remodeling. Clinically, considerable loss of cardiac mass and serological surges of heart failure biomarker NT‐proBNP are typical signs in DCM patients, we therefore measured the heart weight/tibia length (HW/TL) ratio and serum NT‐proBNP levels in these modeling mice. As shown in Figure 6G,H, delivery of DACMs into the murine myocardium significantly reduced the loss of cardiac mass and serum NT‐proBNP levels in DCM progression. Concomitantly, histological examinations suggested overt enlargement of the ventricle chamber and increased cardiac fibrosis in the DCM mice, whereas the acquisition of both the phenotypes was extremely mitigated via tail vain injection of the AAV9‐miR set (Figure 6I,J). In aggregate, these observations suggest that DACMs potently retarded DCM progression to heart failure.

### 3.7 Elicited FOXO3 renders myocardium to sustained apoptosis and autophagy thus exacerbating DCM progression

**FIGURE 7:**
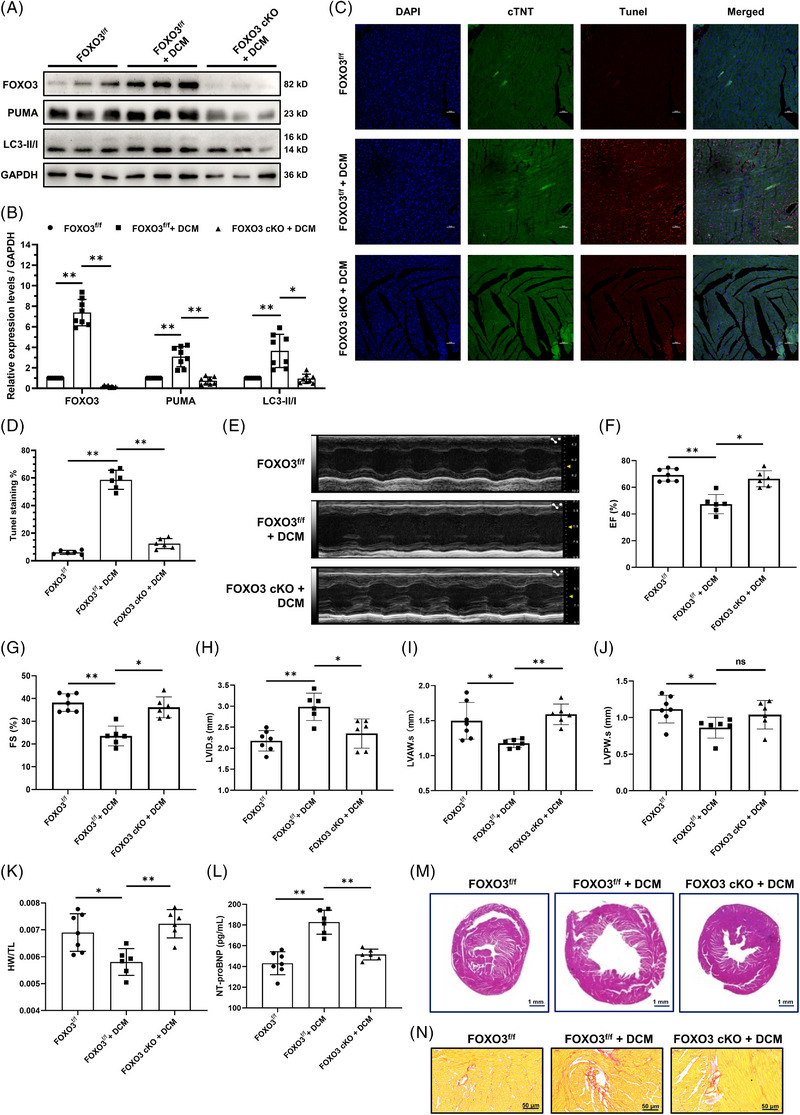
**Cardiac knockout of FOXO3 attenuates myocardial cell apoptosis and autophagy in dilated cardiomyopathy (DCM) progression. (A)** Immunoblotting analysis of the expression of FOXO3, PUMA and LC3‐II/I in the heart tissues from mice specifically knocking out FOXO3 or not during DCM progression. FOXO3^f/f^, control; FOXO3 cKO, cardiac‐specific knockout of FOXO3. GAPDH served as a loading control. (**B)** The density of the signals in A was scanned and plotted as the means ± SD, *n* = 8 for each group. (**C)** TUNEL staining was used to assess murine cardiac apoptosis. Nuclei, DAPI (blue); cardiomyocytes, cardiac troponin T (cTnT) (green); TUNEL (red). Scale bar, 50 μm. (**D)** Quantification of the TUNEL staining in six microscopic fields from three hearts per group in C. (**E)** Representative echocardiographic images of mice described in A. (**F–J)** Echocardiography evaluation for murine left ventricular ejection fraction (F, EF), left ventricular fractional shortening (G, FS), left ventricular internal diameter at end‐systole (H, LVID.s), left ventricular anterior wall thickness at end‐systole (I, LVAW.s), left ventricular posterior wall thickness at end‐systole (J, LVPW.s), *n* = 6–7. (**K)** Statistical analysis of murine heart weight/tibia length (HW/TL) ratio, *n* = 6–7. (**L)** Determination of murine serum NT‐proBNP levels by ELISA, *n* = 6–7. (**M and N)** Representative histological staining images of heart tissue sections from mice described in A. M, hematoxylin‐eosin (H&E) staining, scale bar, 1 mm; N, Picrosirius red staining, scale bar, 50 μm. *p < 0.05; **p < 0.01; ns, no significance.

The transcription factor FOXO3 has been reported to contribute to anti‐hypertrophic signaling in the heart by activating autophagic‐lysosomal and ubiquitin‐proteasomal pathways.^5^, ^38^ Expression of constitutively active mutant of FOXO3 (caFOXO3) in the murine myocardium induces no evident increase of myocyte apoptosis, but reversible cardiac atrophy and dysfunction, which could be normalized within 1 month when shutting off the overexpression in affected mice.^5^, ^39^ Nonetheless, a recent study on a mouse model of laminopathies pointed out that suppression of cardiac FOXO3 improved myocardial cell apoptosis and partially rescued DCM phenotypes of the laminopathies.^4^ To corroborate the role of FOXO3 in cardiomyocyte apoptosis and autophagy involved in DCM progression, transgenic mice with inducible cardiac‐specific knockout of the FOXO3 gene (Myh6‐Cre^ERT2^, FOXO3 flox**^+/+^**) were engineered before Dox administration (Figure S6A). At the baseline level, cardiac deprivation of FOXO3 did not alter murine cardiac function and heart morphology (Figure S6B–G). Consistent with the aforementioned observations, the expression levels of FOXO3, PUMA and LC3II/I were all increased in DCM murine heart tissues, whereas FOXO3 knockout dramatically decreased their elevations (Figure 7A,B), indicating that FOXO3 plays a central role in mediating cardiac apoptosis and autophagy in DCM pathogenesis. Furthermore, loss of FOXO3 markedly decreased the TUNEL staining intensity of DCM murine myocardium (Figure 7C,D), reiterating an indispensable contribution FOXO3 made to the exacerbation of myocardial cell apoptosis in the development of DCM. Data from an echocardiographic evaluation suggested that the absence of cardiac FOXO3 significantly ameliorated the aberrant motion of the left ventricular wall (Figure 7E), improved cardiac function (Figure 7F,G), reduced the enlargement of the ventricle (Figure 6H) and delayed ventricular wall thinning in DCM progression (Figure 7I,J). Subsequently, we determined the HW/TL and serum NT‐proBNP levels in these mice. As shown in Figure 7K,L, knockout of FOXO3 significantly suppressed both cardiac mass loss and serum NT‐proBNP augmentation manifested in the DCM mice. Morphological staining further verified the effect of FOXO3 deficiency in establishing a normal DCM chamber size and maintaining the thickness of ventricular wall (Figure 7M), consistent with the echocardiography data. Additionally, a marked decrease in cardiac fibrosis was identified in the myocardium of DCM mice lacking FOXO3 (Figure 7N). Altogether, these results demonstrated that knocking out cardiac FOXO3, the expression of which was prominently induced in DCM, led to a profound decrease in cardiomyocyte apoptosis and autophagy and largely prevented DCM development.

### 3.8 Disruption of the interplay between DACMs and FOXO3 mRNA fails to safeguard against DCM

**FIGURE 8:**
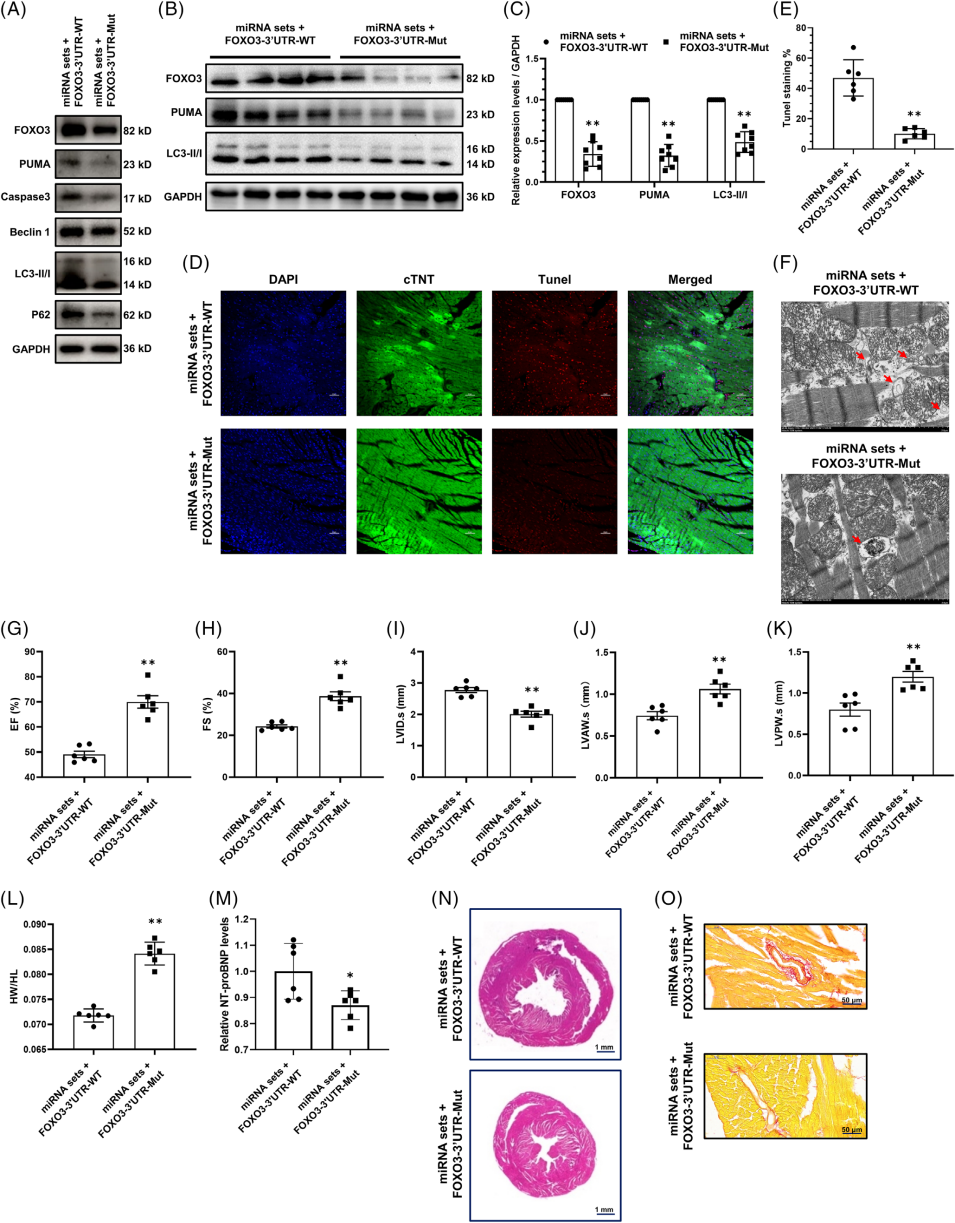
**Disruption of the interplay between dilated cardiomyopathy (DCM)‐associated circulating miRNAs (DACMs) and FOXO3 mRNA fails to safeguard against DCM. (A)** Immunoblotting analysis of the expression levels of FOXO3, PUMA, Caspase3 (cleaved), Beclin1, LC3‐II/I and P62 in Dox‐induced AC16 cells, which were co‐transfected with DACM mimics and constructs expressing FOXO3‐3′UTR‐WT or FOXO3‐3′UTR‐Mut transcript prior to Dox administration. GAPDH served as a loading control. (**B)** Immunoblotting analysis of the expression levels of FOXO3, PUMA and LC3‐II/I in the heart tissues of DCM mouse models, which were generated by co‐infection with AAV9‐miRNA sets and AAV9‐FOXO3‐3′UTR‐WT or AAV9‐FOXO3‐3′UTR‐Mut before DCM induction. GAPDH served as a loading control. (**C)** The density of the signals in B was scanned and plotted as the means ± SD, *n* = 10 for each group. (**D)** TUNEL staining was used to assess murine cardiac apoptosis. Nuclei, DAPI (blue); cardiomyocytes, cardiac troponin T (cTnT) (green); TUNEL (red). Scale bar, 50 μm. (**E)** Quantification of the TUNEL staining in 6 microscopic fields from three hearts per group in D. (**F)** Representative transmission electron microscopic images of the murine heart tissue sections. Red arrow indicated the autophagosome. Scale bar, 2 μm. (**G–K)** Echocardiography evaluation for murine left ventricular ejection fraction (G, EF), left ventricular fractional shortening (H, FS), left ventricular internal diameter at end‐systole (I, LVID.s), left ventricular anterior wall thickness at end‐systole (J, LVAW.s), left ventricular posterior wall thickness at end‐systole (K, LVPW.s), *n* = 6 for each group. (**L)** Statistical analysis of murine heart weight/tibia length (HW/TL) ratio, *n* = 6 for each group. (**M)** Determination of murine serum NT‐proBNP levels by ELISA, *n* = 6 for each group. (**N and O)** Representative histological staining images of heart tissue sections from mice described in B. N, hematoxylin‐eosin (H&E) staining, scale bar, 1 mm. O, Picrosirius red staining, scale bar, 50 μm. *p < 0.05; **p < 0.01.

To elaborate on the role of DACMs‐FOXO3 regulatory axis in DCM pathogenesis, a construct expressing RNA transcript bearing a wild‐type 3′ untranslated region (3′UTR) of FOXO3 gene (wild‐type, FOX3‐3′UTR‐WT) was generated to competitively disrupt the interaction between DACMs and the 3′UTR of endogenous FOXO3 mRNA. Moreover, mutations of seed regions paring with the three DACMs were simultaneously introduced to generate a mutant construct (mutant, FOXO3‐3′UTR‐Mut). According to these devised constructs (Figure S7A), three primer sets capable of producing three different amplicons (FOXO3‐F and FOXO3‐Common‐R for the amplicon FOXO3‐3′UTR‐Common; FOXO3‐F and FOXO3‐WT‐R for the amplicon FOXO3‐3′UTR‐WT; FOXO3‐F and FOXO3‐Mut‐R for the amplicon FOXO3‐3′UTR‐Mut) were designed to specifically determine their expression levels. DACMs were co‐transfected with FOXO3‐3′UTR‐WT or FOXO3‐3′UTR‐Mut into AC16 cardiomyocytes, followed by the treatment of Dox. As shown in Figure S7B,C, DACMs or the exogenously introduced RNA transcripts (FOX3‐3′UTR‐WT and FOX3‐3′UTR‐Mut) were expressed at a comparable level between groups. Under this circumstance, a marked increase in protein expressions of FOXO3, PUMA, Caspase3 (cleaved), Beclin1, LC3‐II/I, and P62 was noted in cells transfected with FOXO3‐3′UTR‐WT, as comparing to that in cells transfected with FOXO3‐3′UTR‐Mut (Figure 8A). This result suggested that the wild‐type FOXO3 3′UTR transcript severely impaired the protection conferred by DACMs against Dox‐provoked cell apoptosis and autophagy; however, the mutant one did not exert the same effect. FOXO3‐3′UTR‐WT and FOXO3‐3′UTR‐Mut expression cassettes were then encapsulated in AAV9 viruses driven by the cTnT promoter. Mice were infected prior to DCM induction with AAV9‐miRNA set together with AAV9‐FOXO3‐3′UTR‐WT or AAV9‐FOXO3‐3′UTR‐Mut (Figure S7D). Likewise, a comparable expression level of DACMs or exogenous RNA transcripts was identified between groups (Supplementary Figure 7E,F). Clearly, FOXO3‐3′UTR‐WT stymied the DACM‐produced repressive effect on the protein expression of FOXO3, PUMA and LC3‐II/I in DCM murine hearts (Figure 8B,C). Moreover, myocardial apoptosis and autophagy were assessed by TUNEL staining and electron microscopic imaging, respectively. We found that FOXO3‐3′UTR‐WT successfully minimized the suppression of DACMs on cardiac apoptosis (Figure 8D,E) and autophagy (Figure 8F) in DCM progression, but FOXO3‐3′UTR‐Mut failed to do that. Additionally, in contrast with the mutant transcript, the wild‐type FOXO3 3′UTR transcript seriously crippled DACMs‐conferred multiple cardioprotective potencies, including cardiac function improvement (Figure 8G,H,M ), heart weight (Figure 8L) and morphology maintenance (Figure 8I–K,N), and cardiac fibrosis reduction (Figure 8O). Collectively, disruption of the interplay between DACMs and FOXO3 mRNA led to failed cardioprotection by DACMs against DCM formation, underscoring the importance of the DACM‐FOXO3 axis in maintaining cardiac homeostasis.

## 4 DISCUSSION

Loss of cardiac miRNA‐mediated regulation leads to mouse DCM and heart failure.^15^, ^16^ An altered miRNA expression pattern has also been noted in human heart tissues procured from different heart diseases, including ischemic cardiomyopathy, DCM, aortic stenosis and systolic heart failure.^13^, ^14^ These discoveries strongly indicate that miRNA plays a pivotal role in DCM development, whereas detailed mechanisms accounting for the pathology need to be further addressed. For a long time, discovery studies have focused on miRNA effects within one specific organ. However, intracellular miRNAs have been shown to cross the membrane barrier, either via encapsulation into membraneous vesicles or by associating with RNA‐binding proteins or lipoprotein complexes, as has been previously described in various human body fluids, including blood, serum/plasma, urine and breast milk.^40^ It has become evident that the informative content can be transmitted and propagated between adjacent and distant cells via extracellular miRNAs. Moreover, the expression patterns of miRNAs in body fluids are highly correlated with disease states and conditions.^17^, ^41^ In this setting, profiling circulating miRNAs, in particular those acting profoundly on cardiac function, not only provides insights useful for establishing next‐generation non‐invasive prognostic and diagnostic applications for heart diseases but also adds new dimensions to understanding the regulatory role played by miRNAs in the heart.

Our work here led us to define a circulating miRNA combination, consisting of miR‐26a‐5p, miR‐30c‐5p, miR‐126‐5p and miR‐126‐3p, that appears closely associated with DCM pathology as evidenced by a dramatic reduction in DCM serum as well as in the heart. Mechanistically, these DCM‐associated circulating miRNAs (DACMs) except miR‐26a‐5p repressed the expression of the common target gene FOXO3, and this combined action was verified to occur within myocardial cells. DACM deficiency‐relieved suppression led to sustained high expression of FOXO3, thereby aggravating cellular apoptosis and autophagy in the myocardium of DCM.

FOXO3 promotes apoptosis and autophagy by trans‐activating apoptosis‐ and autophagy‐associated genes.^42^ PUMA has been proposed to be a central mediator downstream of FOXO3 to motivate cellular apoptosis.^34^, ^36^ In line with this, the expression pattern of FOXO3 demonstrated identical to that of PUMA in cardiomyocyte apoptosis (Figures 5, 7 and 8A‐C), confirming the importance of FOXO3/PUMA axis in governing cardiac apoptosis of DCM. It is worth noting that cardiac‐specific expression of constitutively active FOXO3 (caFOXO3) alone appears unable to induce a marked death of myocardial cells in the murine heart.^5^, ^39^ Albeit with this finding, FOXO3 was proven bona fide as an effective target for repressing myocardial cell apoptosis of the familial DCM identified in laminopathies.^4^ This could be explained by the fact that apoptosis initiation is actually determined by the balance between pro‐apoptotic and anti‐apoptotic gene expression. In this regard, FOXO3 renders cardiomyocytes susceptible to apoptosis stimuli, particularly with respect to DCM risk factors such as genetic perturbations, ischemic injuries, myocarditis and chemical drugs but does not directly trigger cellular apoptosis.

In contrast, FOXO3 poises cells for the rapid induction of autophagy by activating the transcription of autophagy initiator genes, including LC3B, Bnip3 and Beclin1.^25^, ^33^, ^35^ Interestingly, FOXO3 itself has been reported to be a substrate for basal autophagic degradation.^43^ When autophagic flux is blocked by either pharmacological inhibition or genetic deletion of essential autophagy genes, the increased FOXO3 translocates to the nucleus where it can trans‐activate target genes, attempting to compensate for the perturbation in autophagy, and on the other hand sensitizing cellular apoptosis. As such, FOXO3 acts as a cell surveillance mechanism to correct autophagy aberrances, and confers apoptosis sensitization if the autophagy imbalance is not rectified.^23^ According to this rationale, FOXO3 elevation observed in the cultured cardiomyocytes receiving Dox treatment (Figure 5A) could be partially attributed to the autophagic flux blockade by Dox‐induced malfunction of lysosome acidification.^37^ Nevertheless, introduction of DACMs into cardiomyocytes significantly decreased the induction of FOXO3, reiterating a dominant role DACMs played in regulating FOXO3 expression even on the circumstance of Dox administration. Persistent activation of FOXO3, by either reducing its phosphorylation mediated by PI3K/Akt signaling or enhancing its expression via the release of certain critical braking factors, incites excessive autophagy, and thus reportedly makes great contributions to multiple muscular atrophy diseases.^35^, ^42^ In keeping with this notion, caFOXO3 was found previously to trigger robust increase in cardiomyocyte autophagy concomitant with progressive loss of cardiac mass and severe decrease in heart size and cardiac performance.^5^, ^39^ Similarly, cardiac inactivation of FOXO3 by p8^44^, ^45^ or silencing of FOXO3 via gene knockout (Figure 7A,B) evidently diminished DCM myocardium autophagy and improved cardiac function, corroborating the seditious status of FOXO3 in regulating muscular autophagy and atrophy.

The DCM mouse model in this study was generated via administration of doxorubicin that is known to produce cardiotoxicity^46^ and is controversial for its contribution to cellular autophagy.^47^ Currently, the preponderance of evidence indicates that Dox initiates autophagy while hampering the fusion of autophagosomes and lysosomes, resulting in a block stampede in autophagic flux and accelerating cardiomyocyte death. Presumably, FOXO3 makes substantial contributions to this process due to its accompanied expression pattern (Figure 5A) and versatile roles in both facilitating apoptosis and launching autophagy initiation. Distinct from doxorubicin cardiotoxicity, which leads to negligible changes in ventricular morphometry, doxorubicin cardiomyopathy is progressive and well‐characterized with morphological dilation of ventricles, which is also reflected by the different time durations required for generating models of cardiotoxicity^37^, ^48^ and cardiomyopathy (Figure S2B, S5A, S6A and S7D). Hence, we believe the observed stimulation of cardiac autophagy in doxorubicin‐induced DCM (Figure 5E,F,I), long after cessation of drug administration, is likely secondary to acquisition of the remodeling phenotype, consistent with precedent reports.^37^ Indeed, electron microscopy revealed that autophagic vacuoles were abundant within cardiomyocytes from DCM patients,^49^ confirming the universality of the potentiation of cardiac autophagy in DCM of heterogeneous etiologies.

DACMs were proposed to attenuate the intensification of both cardiac apoptosis and autophagy by repressively acting on FOXO3 mRNA, a finding strongly supported by the observations after competitive disruption of the interaction between DACMs and FOXO3 mRNA using the ectopically introduced transcript of FOXO3 3′UTR (Figure 8A‐F). Since DACM‐mediated suppression of FOXO3 occurs within cardiomyocytes across species, and DACM deficiency has been identified in the circulation of DCM subjects regardless of etiology, probably DACM‐FOXO3 axis in governing cardiomyocyte apoptosis and autophagy could be extrapolated to diverse forms of DCM pathogenesis.

## 5 CONCLUSION

**FIGURE 9:**
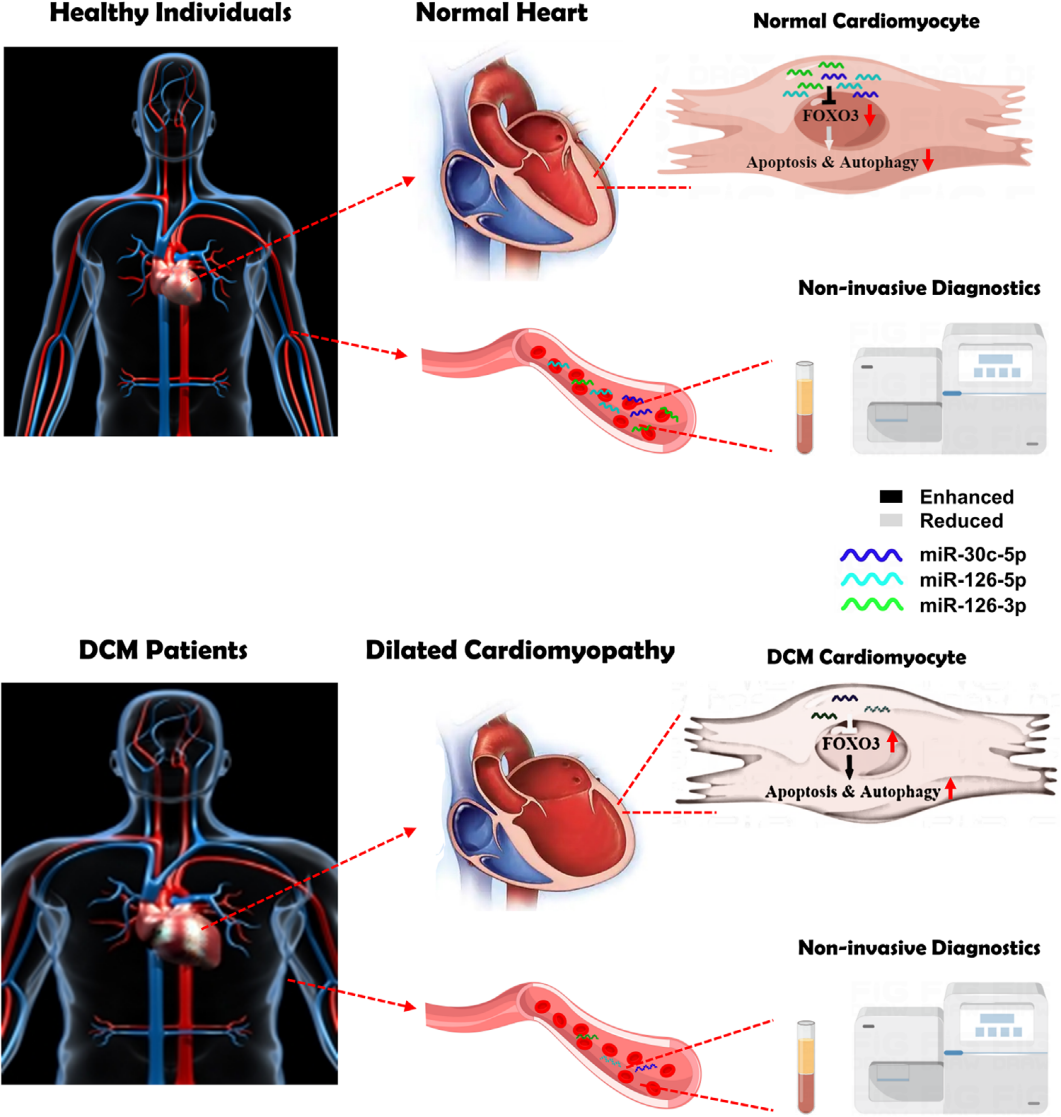
**Schematic description of the major findings on circulating microRNAs closely associated with the development of dilated cardiomyopathy**.

Our study here points out that circulating cardiac miRNA‐FOXO3 axis plays a crucial part in safeguarding against myocardial apoptosis and excessive autophagy, thereby preventing DCM and maintaining cardiac homeostasis. Unambiguously, these findings furnish DCM with serological cues for non‐invasive diagnosis in the future and shed new light on DCM pathogenesis and associated therapeutic targets (Figure 9).

## CONFLICT OF INTEREST STATEMENT

Lin Liu, Hao Tang and Junyue Xing have filed a patent regarding the diagnostic and therapeutic use of microRNAs in DCM. The remaining authors declare that they have no known competing financial interests or personal relationships that could have appeared to influence the work reported in this paper.

## Supporting information

Supporting Information
