# Supplementary material for: Circulating cardiac MicroRNAs safeguard against dilated cardiomyopathy
Source: Clin Transl Med. 2023 May 3;13(5):e1258. doi: 10.1002/ctm2.1258 (PMC10157268; doi:10.1002/ctm2.1258)
Supplement: Supplementary file 1 — Supporting Information [file CTM2-13-e1258-s001.docx]

**Supplementary Figures and Supplementary Tables**

**Supplementary Figure 1 Characterization of the dilated cardiomyopathy (DCM).**

**A - C** Echocardiographic evaluation of DCM patients (DCM) and healthy control persons (Control). Comparing to Control group, V-TR, LAVI, MV-E/e', EDV, ESV and GWW values increased significantly, while MV-e, MV-e', SV, EF, GWI, GCW, and GWE values were shown to be reduced in DCM group. V-TR, velocity of tricuspid regurgitation; LAVI, left atrium volume index; MV-E/e’, ratio of the peak mitral flow velocity (MV-E) to the average velocity of the mitral annulus (MV-e'); EDV, end-diastolic volume; ESV, end-systolic volume; SV, stroke volume; EF, ejection fraction; GWI, global work index; GCW, global constructive work; GWW, global work waste; GWE, global work efficiency. **D** Correlation analysis of serum microRNA (miRNA) sequencing data. **P* < 0.05, ***P* < 0.01.

**Supplementary Figure 2 The heart expression changes of DACMs (DCM-associated circulating microRNAs) between DCM and control subjects demonstrate consistent with their circulating counterparts.**

**A** Schematic illustration for the generation of DCM mouse model. Dox, doxorubicin; Echo., evaluation by echocardiography; *i.v.*, intravenous injection via tail. **B** Sequence conservation analysis of the four miRNAs selected from the cohort screening. **C** Reanalysis of the publicly available miRNA data from human failing and healthy control hearts.

**Supplementary Figure 3 Determination of the overexpression of involved microRNAs.**

**A - D** Relative microRNA expression levels assessed by quantitative polymerase chain reaction (qPCR) in AC16 cells corresponding to each group in Figure 2A (A), 2D (B), 2E (C), and 2F - 2H (D). Data represent as mean ± SD from 3 independent experiments. ***P* < 0.01.

**Supplementary Figure 4 DACMs repress cardiomyocyte expression of FOXO3.**

**A** Purity check for the isolation of cardiomyocytes (CMs) and non-cardiomyocytes (Non-CMs) by quantitative polymerase chain reaction (qPCR). COL1A1, a marker gene for Non-CMs; cTnT, a marker gene for CMs. **B** Representative immunofluorescence images of liver, kidney and heart tissue sections from mice injected with adeno-associated virus serotype 9 (AAV9) to verify the infection specificity and efficiency. AAV9, GFP (green). Scale bar, 100 µm. **C** Relative expression levels of DACMs in Langendorff-isolated murine cardiomyocytes by qPCR. ***P* < 0.01.

**Supplementary Figure 5 Myocardial augmentation of DACMs does not affect mouse cardiac** **function at baseline.**

**A** Schematic description for the establishment of mouse models. Dox, doxorubicin; Echo., evaluation by echocardiography; *i.v.*, intravenous injection via tail. **B** Statistical analysis of the heart weight/tibia length (HW/TL) ratio from mice infected with AAV9-Ctrl or AAV9-miR sets viruses, n = 5 - 6. **C** ELISA determination of the serum NT-proBNP levels from mice described in B, n = 5 - 6. **D** Echocardiographic evaluation for the heart function of mice described in B. EF, left ventricular ejection fraction; FS, left ventricular fractional shortening; LVID.d and LVID.s, left ventricular diameter at end-diastole and end-systole; LVAW.d and LVAW.s, left ventricular anterior wall thickness at end-diastole and end-systole; LVPW.d and LVPW.s, left ventricular posterior wall thickness at end-diastole and end-systole. **E** Relative expression levels of DACMs in heart tissues from mice described in Figure 4E, n = 6 for each group. **P* < 0.05, ***P* < 0.01, ns, no significance.

**Supplementary Figure 6 Cardiac-specific knockout of FOXO3 does not affect mouse cardiac function at baseline.**

**A** Schematic description for the generation of mouse models. Dox, doxorubicin; TAM, tamoxifen; Echo., evaluation by echocardiography; *i.v.*, intravenous injection via tail; *i.p.*, intraperitoneal injection. **B** Representative echocardiographic images of control (FOXO3^f/f^) and FOXO3 knockout (FOXO3 cKO) mice. **C - G** Echocardiograpy evaluation for murine left ventricular ejection fraction (C, EF), left ventricular fractional shortening (D, FS), left ventricular diameter at end-systole and end-diastole (E, left, LVID.s; right, LVID.d), left ventricular anterior wall thickness at end-systole and end-diastole (F, left, LVAW.s; right, LVAW.d), left ventricular posterior wall thickness at end-systole and end-diastole (G, left, LVPW.s; right, LVPW.d), n = 6 for each group. ns, no significance.

**Supplementary Figure 7 Evaluation of the expression of DACMs and 3’ untranslated region (3’UTR) transcript derived from human FOXO3 mRNA.**

**A** Schematic depiction of the primer design strategy for the evaluation of exogenously introduced FOXO3 3’UTR transcript. **B** Relative expression levels of DACMs assessed by quantitative polymerase chain reaction (qPCR) in AC16 cells corresponding to each group in Figure 7A. Data represent as mean ± SD from 4 independent experiments. **C** Relative expression levels of FOXO3 3’UTR transcripts assessed by reverse transcription-polymerase chain reaction (RT-PCR) in AC16 cells described in Figure 7A. GAPDH served as a loading control. **D** Schematic description for the generation of mouse models. Dox, doxorubicin; Echo., evaluation by echocardiography; *i.v.*, intravenous injection via tail. **E** Relative expression levels of DACMs assessed by qPCR in the heart tissues from mice described in Figure 7B, n = 6 for each group. **F** Relative expression levels of FOXO3 3’UTR transcripts assessed by RT-PCR in the heart tissues from mice described in E. GAPDH served as a loading control. ns, no significance.

**Table S1 Twelve microRNAs obtained by cross-referencing the differentially expressed circulating microRNAs with previously documented cardiac microRNAs.**

**Table S2 MiEAA - microRNA enrichment and annotation - analysis results.**

**Table S3 Primer pairs used in this study.**

**Table S1 Twelve microRNAs obtained by cross-referencing the differentially expressed circulating microRNAs with previously documented cardiac microRNAs.**

| Gene Symbol | Type | log2 (DCM / Control) | Q-value (DCM / Control) |
| --- | --- | --- | --- |
| hsa-let-7d-5p | miRNA | -6.585871494 | 2.07E-05 |
| hsa-let-7f-5p | miRNA | -8.161775095 | 2.19E-08 |
| hsa-miR-126-3p | miRNA | -9.364297982 | 3.16E-44 |
| hsa-miR-126-5p | miRNA | -14.96180011 | 7.07E-28 |
| hsa-miR-133a-3p | miRNA | -2.226068079 | 0.004512797 |
| hsa-miR-24-3p | miRNA | -6.953114783 | 2.37E-07 |
| hsa-miR-26a-5p | miRNA | -8.789138864 | 1.67E-06 |
| hsa-miR-26b-3p | miRNA | -8.76763273 | 8.95E-04 |
| hsa-miR-27b-3p | miRNA | -7.959609447 | 2.09E-06 |
| hsa-miR-27b-5p | miRNA | -16.82997043 | 0.004788205 |
| hsa-miR-30c-5p | miRNA | -13.26224169 | 1.41E-10 |
| hsa-miR-451a | miRNA | -1.800598668 | 0.003235319 |

**Table S2 MiEAA - microRNA enrichment and annotation - analysis results.**

| **Category** | | **Subcategory** | **P-adjusted** | | **Observed** | **miRNAs/precursors** |
| --- | --- | --- | --- | --- | --- | --- |
| Target genes (miRTarBase) | FOXO3 | | 0.0170176 | | 4 | hsa-miR-26a-5p; hsa-miR-30c-5p; hsa-miR-126-5p; hsa-miR-126-3p |
| Target genes (miRTarBase) | PTPN7 | | 0.0170176 | | 2 | hsa-miR-126-5p; hsa-miR-126-3p |
| Target genes (miRTarBase) | ZNF506 | | 0.0170176 | | 2 | hsa-miR-26a-5p; hsa-miR-30c-5p |
| Target genes (miRTarBase) | ABCB9 | | 0.0242349 | | 2 | hsa-miR-26a-5p; hsa-miR-24-3p |
| Target genes (miRTarBase) | ADAM9 | | 0.0242349 | | 3 | hsa-miR-30c-5p; hsa-miR-126-5p; hsa-miR-126-3p |
| Target genes (miRTarBase) | CTGF | | 0.0242349 | | 3 | hsa-miR-26a-5p; hsa-miR-30c-5p; hsa-miR-133a-3p |
| Target genes (miRTarBase) | PER2 | | 0.0242349 | | 4 | hsa-miR-24-3p; hsa-miR-30c-5p; hsa-miR-133a-3p; hsa-miR-27b-5p |
| Target genes (miRTarBase) | RASGRP3 | | 0.0260678 | | 3 | hsa-miR-26a-5p; hsa-miR-24-3p; hsa-miR-30c-5p |
| Target genes (miRTarBase) | DNMT1 | | 0.0339779 | | 3 | hsa-miR-26a-5p; hsa-miR-30c-5p; hsa-miR-126-3p |
| Target genes (miRTarBase) | EPG5 | | 0.0339779 | | 3 | hsa-miR-26a-5p; hsa-miR-24-3p; hsa-miR-30c-5p |
| Target genes (miRTarBase) | MMP7 | | 0.0339779 | | 2 | hsa-miR-126-5p; hsa-miR-126-3p |
| Target genes (miRTarBase) | IQCB1 | | | 0.0363677 | 2 | hsa-miR-24-3p; hsa-miR-30c-5p |
| Target genes (miRTarBase) | KPNA6 | | | 0.0363677 | 4 | hsa-miR-26a-5p; hsa-miR-24-3p; hsa-miR-30c-5p; hsa-miR-133a-3p |
| Target genes (miRTarBase) | MYC | | | 0.0404607 | 4 | hsa-miR-24-3p; hsa-miR-30c-5p; hsa-miR-126-5p; hsa-miR-451a |
| Target genes (miRTarBase) | TKT | | | 0.0404607 | 2 | hsa-miR-26a-5p; hsa-miR-30c-5p |
| Target genes (miRTarBase) | NFKBIA | | | 0.0473408 | 2 | hsa-miR-24-3p; hsa-miR-126-3p |

**Table S3 Primer pairs used in this study.**

| Primer name | Sequence |
| --- | --- |
| miRNA-specific RT primer | CAGGTCCAGTTTTTTTTTTTTTTTVN |
| miR26a-5p | GCAGTTCAAGTAATCCAGGATAG |
|  | GGTCCAGTTTTTTTTTTTTTTTAGC |
| miR30c-5p | GCGCAGTGTAAACATCCTAC |
|  | CCAGTTTTTTTTTTTTTTTGCTGAGA |
| miR126-5p | CGCAGCATTATTACTTTTGGT |
|  | CCAGTTTTTTTTTTTTTTTCGCGTA |
| miR126-3p | GCAGTCGTACCGTGAGT |
|  | TCCAGTTTTTTTTTTTTTTTCGCA |
| miR133a-3p | TGGTCCCCTTCAACCAG |
|  | GGTCCAGTTTTTTTTTTTTTTTCAG |
| miR27b-3p | GCAGTTCACAGTGGCTAAG |
|  | TCCAGTTTTTTTTTTTTTTTGCAGA |
| miR24-3p | AGTGGCTCAGTTCAGCA |
|  | CCAGTTTTTTTTTTTTTTTCTGTTCCT |
| miR23b-3p | CAGATCCATTGCCAGGGA |
|  | GTCCAGTTTTTTTTTTTTTTTGGTA |
| miR208a-5p | GCTTTTGGCCCGGGTT |
|  | CAGGTCCAGTTTTTTTTTTTTTTTGTA |
| U6 | CTCGCTTCGGCAGCACA |
|  | AACGCTTCACGAATTTGCGT |
| GAPDH | TGCGACTTCAACAGCAACTC |
|  | ATGTAGGCAATGAGGTCCAC |
| FOXO3-3’UTR-WT | CTTGGTACCGAGCTCGGATC |
|  | CGTCCCATAAACCATCGCAAT |
| FOXO3-3’UTR-Mut | CTTGGTACCGAGCTCGGATC |
|  | AGCACGGATATACCTACGCTT |
| FOXO3-3’UTR-Common. | CTTGGTACCGAGCTCGGATC |
|  | GGCTCACCACCCTGTACAAG |

**Detailed Materials and Methods**

**Human serum and heart tissue samples**

Human serum (10 DCM patients vs. 10 healthy control persons) prepared for microRNA (miRNA) sequencing and the larger cohort serum samples (54 DCM patients vs. 46 healthy control persons) utilized for the validation of circulating miRNA expression changes were procured in Fuwai Central China Cardiovascular Hospital. Patients with established dilated cardiomyopathy were recruited from heart failure clinics at Fuwai Central China Cardiovascular Hospital. The diagnosis of DCM is in accordance with the Chinese Guidelines for the Diagnosis and Treatment of Dilated Cardiomyopathy (2018). The clinical criteria are the objective evidence of ventricular enlargement and decline of myocardial systolic function: 1) left ventricular end-diastolic diameter (LVEDd) > 5.0cm (female), LVEDd > 5.5cm (male) (or greater than 117% of the predicted value of age and body surface area, that is, twice the predicted value of SD + 5%); 2) LVEF < 45% (Simpsons method), LVFS < 25%; 3) Excluding hypertension, valvular heart disease, congenital heart disease or ischemic heart disease. Some additional exclusion criteria were as follows: age less than 18 years, cachexia, combined with rheumatic immune system diseases, vasculitis, myocarditis, pulmonary hypertension and thyroid dysfunction. The control group was matched and screened from healthy people in physical examination according to age, sex, etc. Human myocardial samples used for RNAscope-based RNA *in-situ* hybridization analysis were collected from patients with hypertrophic obstructive cardiomyopathy who underwent modified Morrow myectomy procedures in Fuwai Central China Cardiovascular Hospital. This study was approved by the local ethics board (2019042) in accordance with the Declaration of Helsinki. All participants signed written consent for the study investigations.

**Animal Model of dilated Cardiomyopathy**

C57BL/6 mice (7 - 8 weeks old, male and female for fifty-fifty) purchased from the Institute of Laboratory Animal Science, Chinese Academy of Medical Sciences (Beijing, China), were maintained on a 12-hour light/dark cycle from 6 am to 6 pm. Mice were adaptive feeding for 1 week and were conducted subsequent experiments. To specifically overexpress miRNA-30c-5p miRNA-126a-5p and miRNA-126-3p in the myocardium, mouse precursor miRNA-30c and miRNA-126a was simultaneously constructed in one vector under the control of the cardiac cardiac troponin T (cTnT) promoter. For specifically overexpression of FOXO3-3’UTR-WT or FOXO3-3’UTR-Mut, related gene fragments and a part of fragment derived from pcDNA vector were simultaneously inserted downstream of the cTnT promoter. Primers used for the amplification of exogenous FOXO3 fragments referred to the following materials. Mice were given a single intravenous injection of an AAV9 vector via the tail vein at a concentration of 1 × 10^11^ viral genomes per mouse. AAV9 viruses used in this study were generated by Hanbio Biotechnology Co. (Shanghai, China). Four weeks post-AAV9 injection, the mice were injected via the tail vein with doxorubicin (Dox, 5 mg/kg, MCE, Cat#HY-15142A) or normal saline (NS) once weekly for 4 weeks to establish DCM model. Cardiac function was examined in acclimatized, unanesthetized mice by echocardiography (Vevo 3000), 12 weeks after the final injection. For cardiomyocyte-specific deletion of FOXO3, Myh6-CRE^ERT2^ mouse was crossed with FOXO3^f/f^ mice. All mouse lines were maintained in a C57BL/6J genetic background. 8 weeks Myh6-CRE^ERT2^-FOXO3^f/f^ mice and the littermate FOXO3^f/f^ mice used as control were injected intraperitoneally with Tamoxifen (30 mg/kg/d, Sigma, Cat#H6278) once every 24 hours for a total of 5 consecutive days. Subsequent procedure was conducted seven days after last injection.

**Langendorff isolation of murine cardiomyocytes**

The detailed methods for the isolation of adult mice cardiomyocytes by the langendorff method was described previously.^1^ Briefly, mice were given intraperitoneal injection of 1000 U/kg heparin before anesthesia, followed by deep anesthesia with isoflurane and sacrificed by cervical dislocation. Cut the thoracic cavity to fully expose the heart, cut off the heart along the root of the aorta, and quickly place it in the ice-cold calcium-free solution to remove the lungs. The aorta is then positioned onto the cannula using fine-tipped forceps and secured with a 6-0 silk suture and then connecting the cannulated heart to the Langendorff apparatus. Perfuse with calcium-free solution for 3 - 5 min, 4 ml/min, and then digest with enzyme digestion buffer (0.67g/L BSA + 83U/ml Collagenase II) for about 15 - 20min, until the heart becomes soft, flabby and pale. Left ventricles were excised and shredded into small pieces in calcium-free solution containing 1 mg/ml BSA and gently dissociated for 1 - 3 min to facilitate myocyte dissociation. Cell suspension was filtered through a 100 mesh-cell strainer and centrifuged at 100g for 1 min. The supernatant was discarded and the cell pellet was resuspended in calcium-free solution. Centrifuged at 100 g for 1 min, and the cell mass was collected to obtain relatively pure cardiomyocytes.

**Cell culture and transfection**

AC16 cells were cultured in Dulbecco’s modified Eagle’s medium (Hyclone) supplemented with 10% fetal bovine serum (Gibco) and antibiotics, at 37°C in 5% CO2. Human iPSC-derived cardiomyocytes (hiPSC-CMs), well differentiated (40 days) and tested in purity and electrophysiological characteristics of the cardiomyocytes, were purchased from Help Stem Cell Innovations Co., Ltd. (Nanjing, China) and maintained in culture following the manufacturer’s instruction. Neonatal rat ventricular myocytes (NRVMs) were isolated and cultured as described previously.^2^ Briefly, the heart of newborn rats of 1 - 3 days old were harvested and rinsed in ice-cold PBS to remove residual blood. After cutting off the atrium and aorta and other tissues, the heart was cut into pieces and added to the digestion buffer (0.125% trypsin + 1 mg/ml Collagenase II). Shake gently in a water bath at 37°C for 10 min, and then transfer the supernatant to a 50 ml tube with equal volume of ice-cold culture medium (DMEM/F12 + 10% FBS + 1% Penicillin/Streptomycin) after natural sedimentation. Repeat the above digestion steps about 4-5 times until the heart tissue was completely digested. All cell suspensions were collected after filtration through a 100 mesh-cell strainer. After centrifugation at 500 g for 5 min, the supernatant was discarded, and the cell pellet was resuspended with culture medium and seeded in a petri dish. After 1-1.5h, the unattached cell suspension was collected, centrifuged at 500g for 5min, and the cell pellet was resuspended in Brdu-containing culture medium (culture medium + 0.1mM Brdu). The cells were inoculated in the culture plate (about 5 × 10^5^ cells/ml), and then placed in a 37°C, 5% CO2 incubator and the new culture medium was replaced after 48h. For transient transfection of miRNA minics or NC, Lipofectamine 2000 reagent (Thermo fisher Scientific, Cat#11668019) was used according to the manufacturer’s protocol. hiPSC-CMs were infected with recombinant adenovirus (rAdV) expressing miRNA mimics or NC (Hanbio Biotechnology Co., Shanghai, China). RFP-GFP-LC3 virus was packed in 293T cell then was transfected to AC16 cell. The cell was induced by Dox (300nM) for 24 hours and fixed with paraformaldehyde, subsequently stained with DAPI and observed in confocal fluorescence microscope.

**MicroRNA sequencing**

The serum was stored at −80 °C prior to RNA extraction. Total serum RNA was extracted from the sample using TRIzol® LS Reagent (Invitrogen) according the manufacturer’s instructions (Invitrogen) and genomic DNA was removed using DNase I (TaKara). Then RNA quality was determined by 2100 Bioanalyzer (Agilent) and quantified using the ND-2000 (NanoDrop Technologies) combined with Qubit Fluorometer (Thermo Fisher). Only high-quality RNA sample (OD260/280 ≥1.0, total amount ≥10 ng, concentration ≥ 2 ng/μL) was used to construct sequencing library. RNA libraries were prepared for sequencing using standard Illumina protocols. RNA-sequencing was performed on BGISEQ-500 platform. The raw sequencing data are called raw tags. The raw tags were processed using the following steps: remove low quality tags; remove tags with 5 primer contaminants; remove tags without 3 primer; remove tags without insertion; remove tags with poly A; remove tags shorter than 18 nt. The clean tags were mapped to the reference genome and other sRNA database including miRbase, siRNA, piRNA and snoRNA with Bowtie2. CMsearch was performed for Rfam mapping. The software miRDeep2 was used to predict novel miRNA by exploring the secondary structure, and Piano was used to predict piRNAs. RNAhybrid miRanda and TargetScan were used to predict target genes of miRNAs. The small RNA expression level is calculated by counting absolute numbers of molecules using unique molecular identifiers. Differential expression analysis was performed using the DEGseq, Q value ≤ 0.001 and the absolute value of Log2Ratio ≥ 1 as the default threshold to judge the significance of expression difference. All target genes were aligned against the Kyoto Encyclopedia of Genes (KEGG) and Gene Ontology (GO) database. GO enrichment analysis and KEGG enrichment analysis of target genes were performed using phyper, a function of R.

**Echocardiography**

Echocardiography measurements were performed at the indicated times to evaluate the cardiac function of the mice. Briefly, mice were anesthetized with 1.5% to 2% isoflurane (ensure that the ventricular rate was 500-600 beats/min) and evaluated by using Vevo 3000 echocardiography (VisualSonics Inc) with a 30 MHz central frequency. Echocardiography data measurements were performed using M-mode images taken from a parasternal short-axis view at the level of the papillary muscles. Averaged LV diastolic and systolic anterior wall thickness (LVAWd, LVAWs), LV diastolic and systolic posterior wall thickness (LVPWd, LVPWs) and LV diastolic and systolic internal dimensions (LVIDd, LVIDs) are measured. Ejection fraction and fractional shortening were calculated as follows: Ejection fraction= (end LV diastolic volume – end LV systolic volume) /end LV diastolic volume × 100%; Fractional shortening= (end LV diastolic diameter – end LV systolic diameter) /end LV diastolic diameter × 100%. The echocardiographer was blinded to the experimental groups.

**RNA Isolation and Polymerase Chain Reaction Analysis**

RNA was isolated from tissues or cultured cells with TRIzol® LS Reagent (Invitrogen) and treated with Superscript II reverse transcriptase (Thermo fisher Scientific, Cat#18090010) with oligo-dT and random hexamers or miRNA-specific RT primer according to the manufacturer’s instructions. U6 was specifically used as control for microRNA normalization, while GAPDH was used for other gene normalization. Real-time qPCR amplification reaction was performed by using Universal SYBR qPCR Master Mix (Vazyme, Cat#Q511-02) and a Bio-Rad CFX96TM Real-time SYSTEM. RT-qPCR analysis was carried out using corresponding primer pairs listed in Table S3.

**Immunoblot Analysis**

Western blot analysis was performed according to the standard procedures. Cells or tissue were lysed with RIPA (adding protease inhibitors), then 20-30ug protein was loaded and fractionated SDS-polyacrylamide gel electrophoresis (SDS-PAGE) and transferred onto nitrocellulose membranes. Afterwards, different antibodies were used to detect expression of respective target, FOXO3 (CST, Cat#2479, 1:1000), p62 (CST, Cat#23214, 1:1000), Beclin1 (CST, Cat#3495, 1:1000), LC3B (CST, Cat#3868, 1:1000), GAPDH (Proteintech, Cat#60004-1-Ig, 1:5000), and cleaved-Caspase3 (CST, Cat#9664, 1:1000) antibodies. Following incubation with the HRP-conjugated secondary antibody, signals were detected by using chemiluminescent western blot detection system (iBright). Intensities of the bands were quantified by Image J software.

**RNAscope**

RNAscope was carried out according to ACD manufacturer’s procedure. Tissue slides were dewaxed, rehydrated, and subjected to antigen retrieval. Then added RNAscope Hydrogen Peroxide and incubated for 10min at room temperature followed by applying RNAscope Protease III at 40 ℃ for 15 min, afterwards proceeding to miRNAscope Assay. Related miR-30c-5p, miR-126a-5p, and miR-126a-3p RNAscope probes were designed by ACD. After probe incubation, Fast red-A and red-B were mixed and added into slide, and incubated at 37℃ for 10 min. Subsequently, the sides were stained with cTnT (Invitrogen, Cat#MA5-12960, 1:200) and counterstained with hematoxylin. Images were captured with Zeiss microscope at 20 × magnification.

**Dual luciferase reporter assays**

AC16 cells were transfected with reporters (pmiRGLO-30c-WT or -Mut, pmiRGLO-126a-5p-WT or -Mut, pmiRGLO-126a-3p-WT or -Mut) using Lipofectamine 3000. The cells were transfected with the pmiRGLO vector as a negative control. 48 hours later, firefly and renilla luciferase activities were measured using dual luciferase detection system (Promega, Cat#E1910) according to the manufacturer’s instructions. Firefly luciferase activity was normalized to Renilla luciferase activity in the same sample. The experiment was repeated three biological times.

**TUNEL staining**

The experiment was conducted following the manufacturer’s procedure (Vazyme, Cat#A113-01). Briefly, tissue sections were dewaxed, rehydrated, and subjected to antigen retrieval. After Proteinase K digested, the sections were stained with TRITC-UTP and terminal transferase (TdT). Subsequently, cTnT was used to indicate cardiomyocyte. The apoptosis index was measured by counting TUNEL-positive puncta in 100 randomly selected cTnT-positive cells in multiple randomly chosen fields at 20 × magnification. Images were captured with a laser-scanning confocal microscope (Zeiss).

**Flow cytometry analysis of apoptosis**

The flow cytometry was used to evaluate cardiomyocyte apoptosis. AC16 was induced by Dox (300nM) for 24 hours and measured with Annexin V-APC/7-AAD kit (BD Biosciences, Cat#E1960). Cells were trypsinized, collected, and resuspended in binding buffer. Cells were added 10 μl of APC-conjugated Annexin V antibody and 5 μl of 7-amino-actinomycin D (7-AAD) and incubated in the dark at room temperature for 15 minutes followed by flow cytometer.

**Serum NT-proBNP measurement**

Mouse blood serum was collected and measured with mouse NT-proBNP elisa kit (Elabscience, Cat#E-EL-M0834c) following the manufacturer’s instructions. All data had obtained from three biological repeats.

**Histology and imaging**

Histology and Imaging measurements were performed at the indicated times. After the mice were fully anesthetized and sacrificed as mentioned above, the right atrium was cut open after the chest cavity was exposed, and the heart was removed after adequate perfusion with 10 ml of normal saline. The hearts were immediately placed in 4% paraformaldehyde and fixed at room temperature for 24 h. After dehydration and embedding, the hearts were serially sectioned along the short axis until the maximum short axis section of the heart was reached. After serial sectioning at 5 μm thickness, HE, Masson, and Picrosirius red staining were performed to evaluate cardiac morphology and degree of fibrosis. The cross-sectional areas of the cardiomyocytes and degree of fibrosis were visualized by a digital scanning imaging system Olympus FV1000 (Olympus, Tokyo, Japan) and quantified by using Image J software (NIH, Bethesda, MD, USA) from captured images of the stained sections.

**Immunofluorescence**

Briefly, myocardial frozen sections were fixed for 10 min in 4% formaldehyde at room temperature. After washed with PBS, sections were blocked in 5% donkey serum with PBST(0.1% Triton X-100) for 1 hour at room temperature and then were incubated with the primary antibodies cTnT (1:200) or FOXO3 (1:200) at 4°C overnight. After washed with PBS, Sections were incubated with relevant secondary antibodies conjugated with fluorescent dyes. The nuclei were stained with 4’,6-diamidine-2’-phenylindole dihydrochloride (DAPI, Sigma Aldrich, Cat#D9542), and the slides were mounted with Permafluor mounting medium (Thermo fisher Scientific,Cat#TA-006-FM). Sections were then examined with an inverted fluorescent microscope (Zeiss). All histologic examinations were performed by an independent observer blinded to the treatment groups and time points.

**Transmission electron microscopy**

Fresh hearts removed from mice were perfused with an electron microscope fixing solution (2.5% glutaraldehyde in 0.1 M phosphate buffer, pH 7.4) and fixed with 2% osmium tetroxide and 0.8% potassium ferrocyanide in a 0.1mol/L sodium cacodylate buffer for 2 h. After washed three times with sodium cacodylate buffer, samples were dehydrated using a density gradient of alcohol and acetone. Finally, the samples were cut into ultrathin sections of 60-80nm, dyed, and dried overnight at room temperature. Images were obtained with an H-7800 TEM (Hitachi High-Technologies Europe GmbH, Krefeld, Germany); acquired images were processed with Digital Micrograph software. Autophagosome were measured at a total of 5 optical fields per heart.

1 Louch, W. E., Sheehan, K. A. & Wolska, B. M. Methods in cardiomyocyte isolation, culture, and gene transfer. *J Mol Cell Cardiol* **51**, 288-298, doi:10.1016/j.yjmcc.2011.06.012 (2011).

2 Liu, C. *et al.* CIRBP-OGFR axis safeguards against cardiomyocyte apoptosis and cardiotoxicity induced by chemotherapy. *Int J Biol Sci* **18**, 2882-2897, doi:10.7150/ijbs.69655 (2022).
